# Supplementary material for: Season, wind speed, and seasonal rain are major drivers of a regional aeolian sediment transport model
Source: PLoS One. 2025 Sep 26;20(9):e0333166. doi: 10.1371/journal.pone.0333166 (PMC12468981; doi:10.1371/journal.pone.0333166)
Supplement: S1 — (PDF) [file pone.0333166.s001.pdf]

DustR Kelvyn Datafull

Kelvyn Bladen

2024-06-24

Contents

Load and Clean Data 2

Data Imputation 5

Plots 6

Models 8

- Random Forest Full . . . 8
- Item 1: Tree Full vs RF Full . . . 11
- Item 2: RF pre15 vs RF Full . . . 15
- Item 3: Moisture analysis . . . 18
- Item 4: Graze and rough analysis . . . 22
- Item 5: Top Variables analysis . . . 25
- Item 6: Rerun full model Samples . . . 28

Comparisons 29

- Item 1: Model . . . 29
- Item 2: Data . . . 29
- Item 3: Moisture . . . 29
- Item 4: Graze and Roughness . . . 30
- Item 5: Top Variables . . . 30
- Item 6: Samples . . . 30
- All R2 values except subsamples . . . 31

Load and Clean Data

```
r2_fun <- function(preds, actual) {
  return(1 - sum((actual - preds) ^ 2) / sum((actual - mean(actual))^2))
}

#datafull2 <- read.csv("newfulldataset.csv", header = TRUE)
datafull2 <- read.csv("my_new_full.csv", header = TRUE)

colnames(datafull2)
```

```
##  [1] "Site"       "Year"       "season"     "elev_type"  "Silt"
##  [6] "Clay"       "C25"        "C50"        "C100"       "C200"
## [11] "B25"        "B50"        "B100"       "B200"       "roughness"
## [16] "mois5"      "mois15"     "mois30"     "condition"  "Elevation"
## [21] "veg"        "sumflux"    "site"       "era"        "seasrain"
## [26] "seasrainr"  "wind"       "avggust"    "maxpeak"    "avgpeak"
## [31] "wind4r"     "wind8r"     "wind12r"    "Sand"       "Bmean"
## [36] "Cmean"      "Cover"      "AggStab"    "BareGround" "BSC"
## [41] "Annuals"
```

```
datafull2 = datafull2 %>% dplyr::arrange(Site, Year, season)

datafull <- datafull2 %>% dplyr::select(!c("site","era"))
colnames(datafull)[22] <- "Sumflux"
datafull$Sumflux <- as.numeric(datafull$Sumflux)
```

```
## Warning: NAs introduced by coercion
```

```
datafull <- datafull %>% dplyr::filter(!is.na(Sumflux))
datafull$Sumflux <- log10(datafull$Sumflux+0.1)

var_list = c(#"Wgust",
             "season", "veg", "seasrain",
             "Sand", "mois5", "mois15","mois30", "condition",
             "B25","B50","B100","B200","roughness",
             "C25","C50","C100","C200",
             "Bmean","Cmean","Cover", "AggStab",
             "BareGround","BSC", "Annuals", # end of varImps
             "Year", "Silt", "Clay", "Elevation", # To try
             "seasrainr", "wind", "avggust",
             "maxpeak", "avgpeak", "wind4r", "wind8r", "wind12r",
             "elev_type", "Site", # drop
             "Sumflux") # response

datafull1 <- datafull[,var_list]

sapply(datafull1, class)
```

```
##      season         veg    seasrain        Sand       mois5      mois15
## "character" "character"   "numeric"   "numeric"   "numeric"   "numeric"
```

```
##      mois30   condition         B25         B50        B100        B200
##   "numeric" "character"   "numeric"   "numeric"   "numeric"   "numeric"
##   roughness         C25         C50        C100        C200       Bmean
##   "numeric"   "numeric"   "numeric"   "numeric"   "numeric"   "numeric"
##       Cmean       Cover     AggStab  BareGround         BSC     Annuals
##   "numeric"   "numeric"   "numeric"   "numeric"   "numeric"   "numeric"
##        Year        Silt        Clay   Elevation   seasrainr        wind
##   "integer"   "numeric"   "numeric"   "numeric"   "numeric"   "numeric"
##     avggust     maxpeak     avgpeak      wind4r      wind8r     wind12r
##   "numeric"   "numeric"   "numeric"   "integer"   "integer"   "integer"
##   elev_type        Site     Sumflux
## "character" "character"   "numeric"
```

```
datafull1$condition <- as.factor(datafull1$condition)
datafull1$elev_type <- as.factor(datafull1$elev_type)
datafull1$veg <- as.factor(datafull1$veg)
datafull1$season <- as.factor(datafull1$season)
datafull1$Site <- as.factor(datafull1$Site)

sapply(datafull1, class)
```

```
##     season        veg   seasrain       Sand      mois5     mois15     mois30
##   "factor"   "factor"  "numeric"  "numeric"  "numeric"  "numeric"  "numeric"
##  condition        B25        B50       B100       B200  roughness        C25
##   "factor"  "numeric"  "numeric"  "numeric"  "numeric"  "numeric"  "numeric"
##        C50       C100       C200      Bmean      Cmean      Cover    AggStab
##  "numeric"  "numeric"  "numeric"  "numeric"  "numeric"  "numeric"  "numeric"
## BareGround        BSC    Annuals       Year       Silt       Clay  Elevation
##  "numeric"  "numeric"  "numeric"  "integer"  "numeric"  "numeric"  "numeric"
##  seasrainr       wind    avggust    maxpeak    avgpeak     wind4r     wind8r
##  "numeric"  "numeric"  "numeric"  "numeric"  "numeric"  "integer"  "integer"
##    wind12r  elev_type       Site    Sumflux
##  "integer"   "factor"   "factor"  "numeric"
```

```
colnames(datafull1)
```

```
##  [1] "season"     "veg"        "seasrain"   "Sand"       "mois5"
##  [6] "mois15"     "mois30"     "condition"  "B25"        "B50"
## [11] "B100"       "B200"       "roughness"  "C25"        "C50"
## [16] "C100"       "C200"       "Bmean"      "Cmean"      "Cover"
## [21] "AggStab"    "BareGround" "BSC"        "Annuals"    "Year"
## [26] "Silt"       "Clay"       "Elevation"  "seasrainr"  "wind"
## [31] "avggust"    "maxpeak"    "avgpeak"    "wind4r"     "wind8r"
## [36] "wind12r"    "elev_type"  "Site"       "Sumflux"
```

```
hist(datafull1$Sumflux)
```

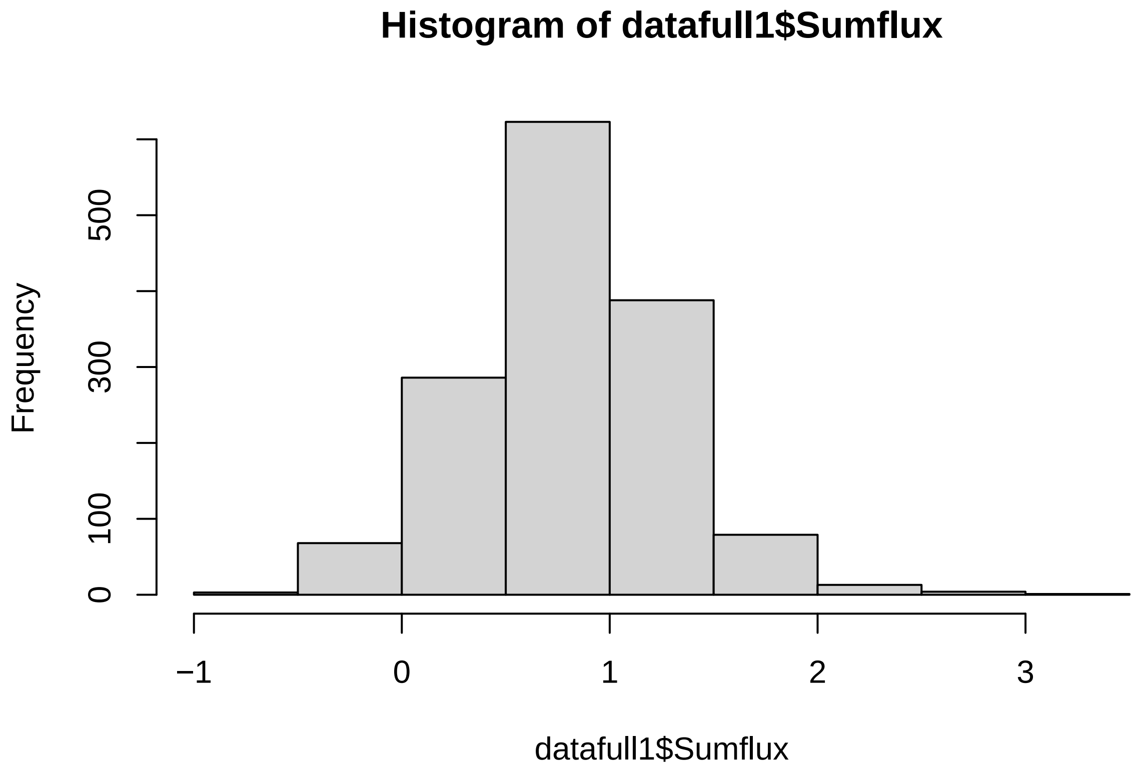

Histogram of datafull1$Sumflux
Frequency
0
100
300
500
−1
0
1
2
3
datafull1$Sumflux

Data Imputation

```
set.seed(100)

sapply(datafull1, FUN = function(x)sum(is.na(x)))

datafull2 <- datafull1 %>% dplyr::select(-Sumflux)
```

```
imp<-mice(datafull2, m=5)
dataimputed = complete(imp)
sapply(dataimputed, FUN = function(x)sum(is.na(x)))
datafull3 <- dataimputed %>% dplyr::select(mois5, mois30, Sand, wind,
                                           wind4r, wind8r, wind12r,
                                           Clay, Silt, Elevation,
                                           season, seasrain)
imp1<-mice(datafull3,m=5)
```

```
dataimputed$mois30 = complete(imp1)$mois30
dataimputed$Sumflux <- datafull1$Sumflux

sapply(dataimputed, FUN = function(x)sum(is.na(x)))

colnames(datafull1)[ncol(datafull1)] <- "logsum"
colnames(dataimputed)[ncol(dataimputed)] <- "logsum"
```

Plots

```
d = datafull1 %>% group_by(season, condition) %>%
  dplyr::summarise(m_flux = 10^mean(logsum)) %>%
  dplyr::arrange(m_flux)
```

```
## `summarise()` has grouped output by 'season'. You can override using the
## `.groups` argument.
```

```
d$season <- factor(d$season, levels = c("Summer","Spring","Winter"))

d$condition <- factor(d$condition,
                      levels = c("No Graze","Grazed","Some Graze"))

d %>% ggplot(aes(x = season, y = m_flux, fill = condition)) +
  geom_bar(position = "dodge", stat = "identity") +
  scale_fill_manual(values = c("No Graze" = "purple",
                               "Grazed" = "springgreen",
                               "Some Graze" = "blue"))
```

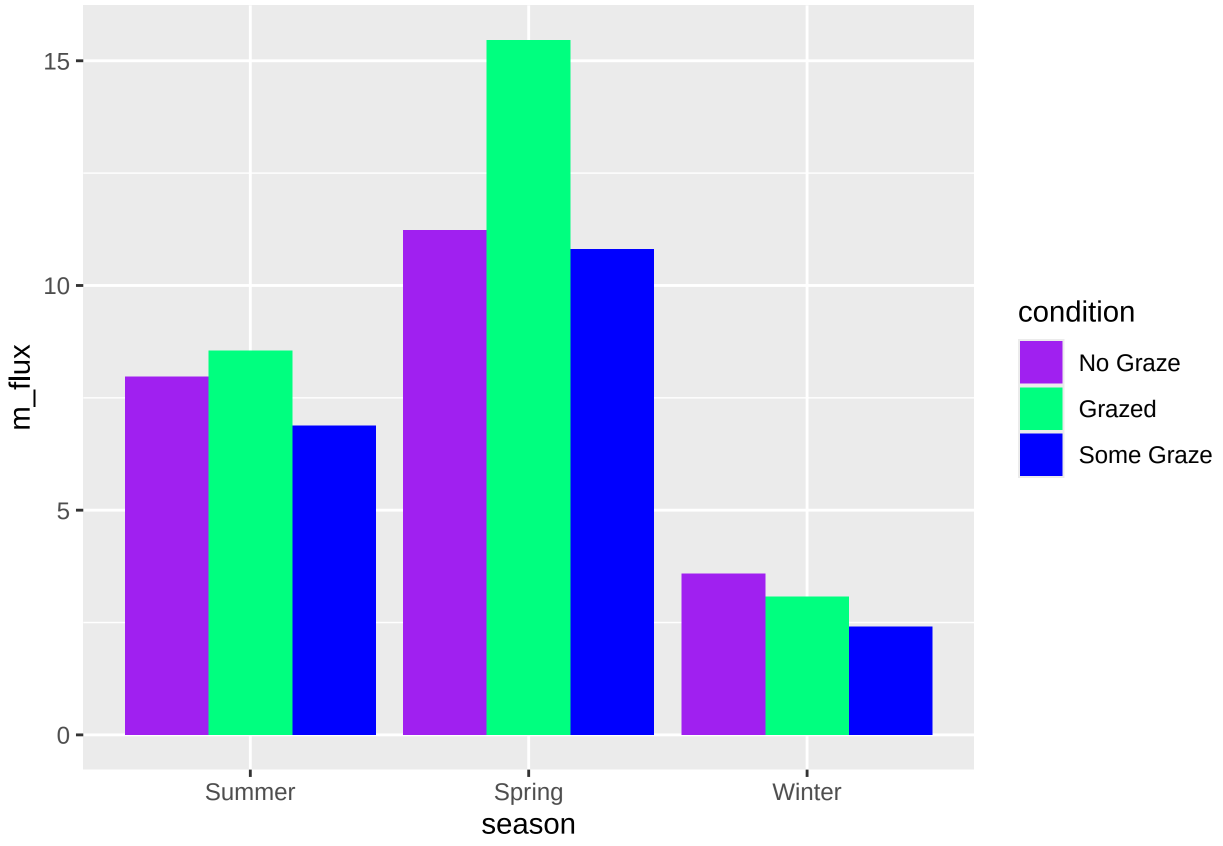


```
#barchart(datafull1$veg)
#datafull4 = datafull1
# datafull4$veg = ifelse(datafull4$veg %in% c("MC","MB"), "Mancos",
```

```
#                              as.character(datafull4$veg))
# datafull4$veg1 = as.factor(datafull4$veg1)

d1 = datafull1 %>% group_by(veg) %>%
  dplyr::summarise(m_flux = mean(10^(logsum))) %>%
  filter(veg %in% c("Blackbrush", "Grassland", "Mancos",
                    "PJ", "Sagebrush", "Saltbush")) %>%
  dplyr::arrange(m_flux)

d1$veg <- factor(d1$veg, levels = d1$veg)

d1 %>% ggplot(aes(x = veg, y = m_flux)) +
  geom_bar(stat = "identity") + ylim(0, 20)
```

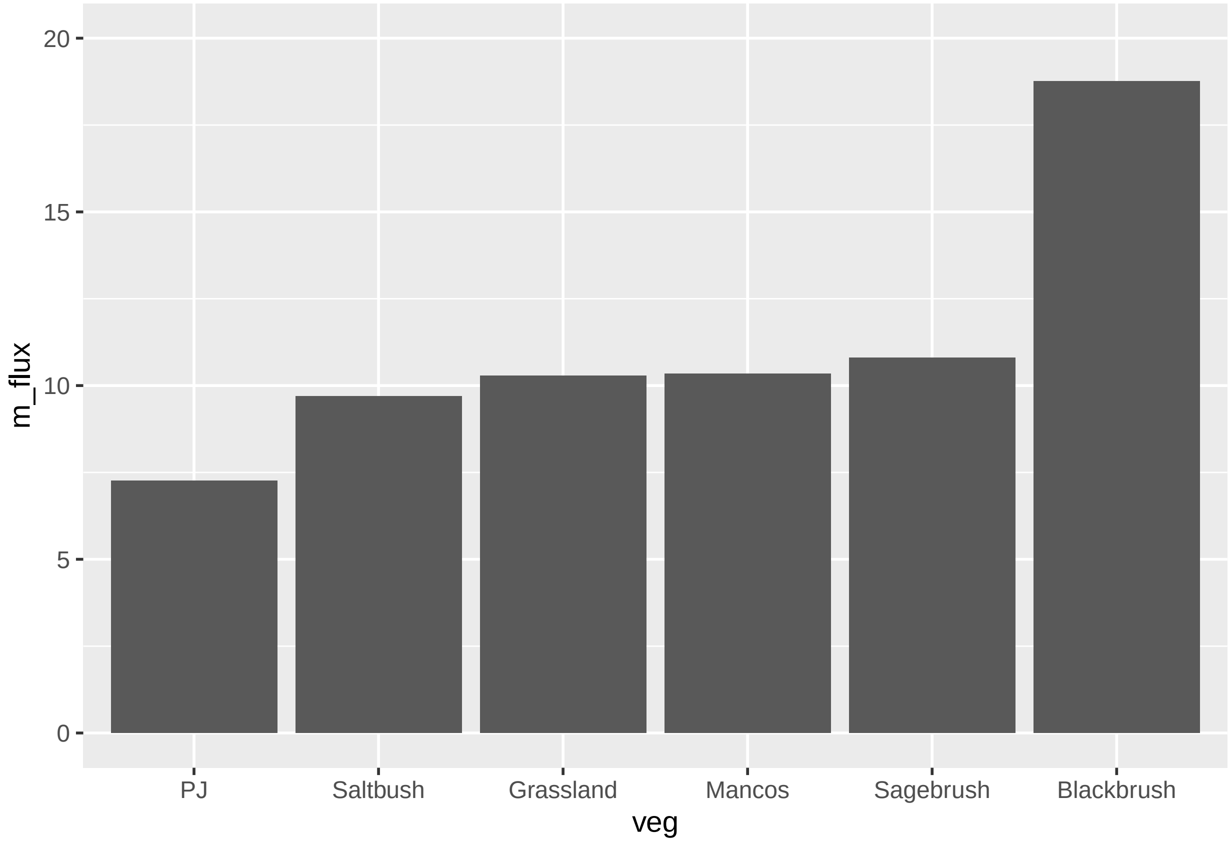


```
datafull <- dataimputed %>% dplyr::select(!c(Site))

data_pre15 <- dataimputed %>% filter(Year < 2015) %>%
  dplyr::select(!c(Site, Year))
```

Models

Random Forest Full

```
set.seed(123)
rf_full <- randomForest(logsum~.,
                        data = datafull, importance = T)
rf_full$rsq[500]
```

```
## [1] 0.6096829
```

```
r2_fun(predict(rf_full, datafull), datafull$logsum)
```

```
## [1] 0.9170689
```

```
randomForestVIP::ggvip(rf_full, sqrt = F, scale = F, num_var = 10)$both_vips
```

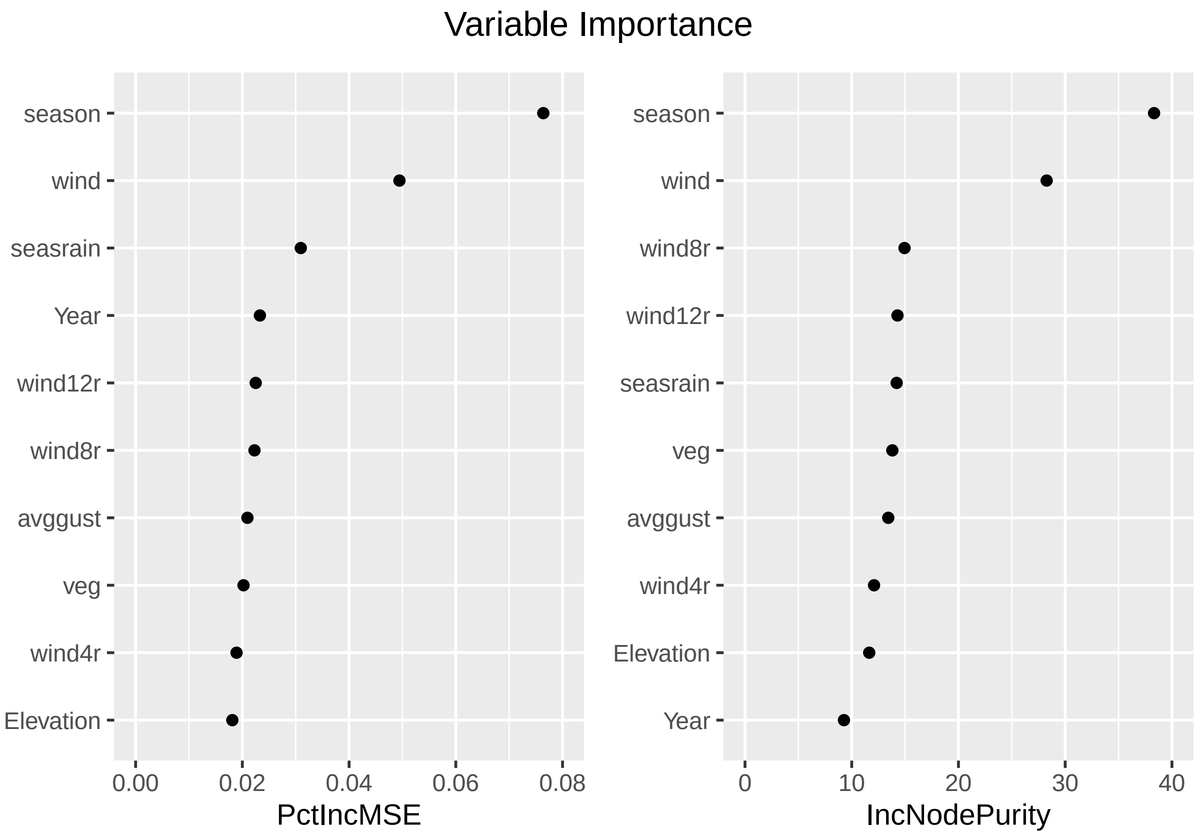


```
#ggsave("var_imp10.pdf", vg, dpi = 1600, width = 3, height = 4)

pd_res <- pdp_compare(rf_full, trellis = F,
                      var_vec = c("seasrain", "season",
                                  "wind", "avggust",
```

```
                                    "wind4r", "wind8r", "wind12r",
                                    "mois5", "mois15","mois30"))
pd_res$full_num
```

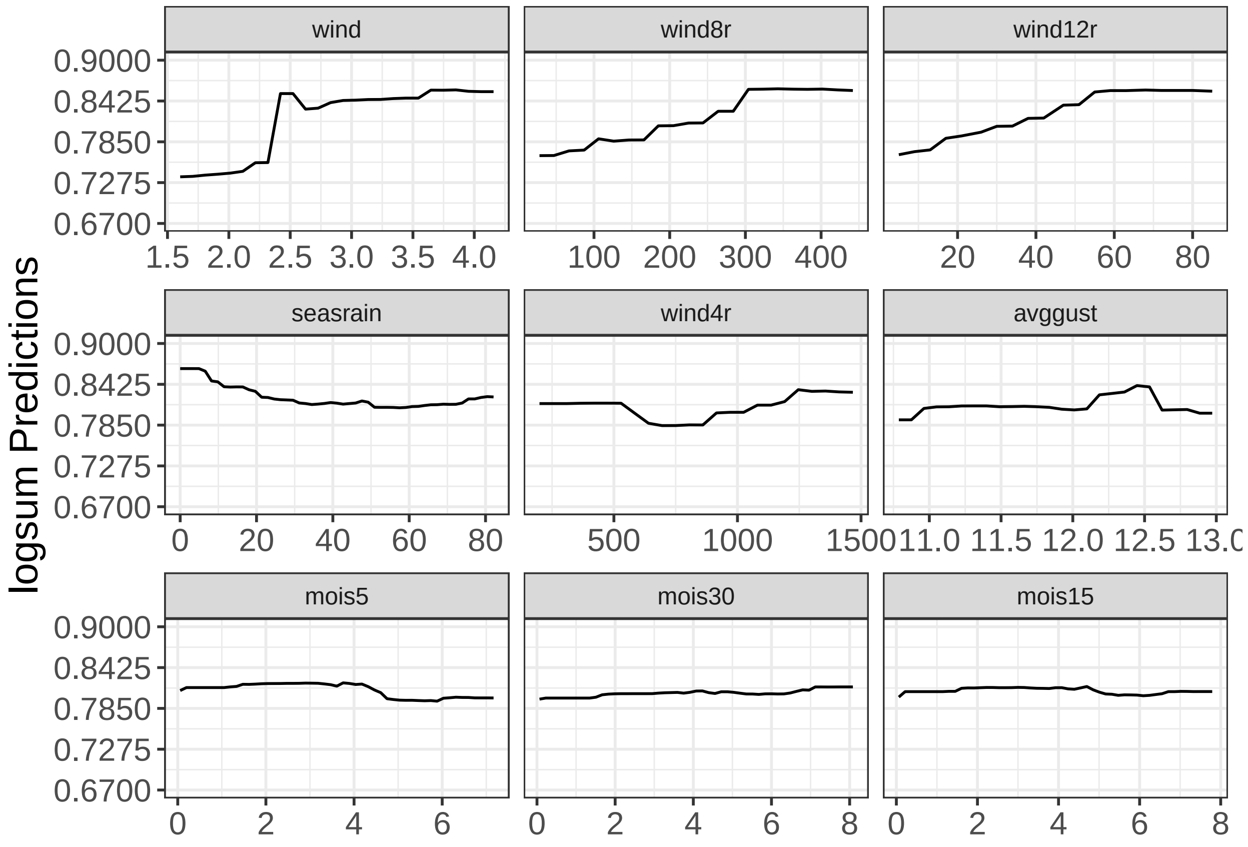


```
pd_res$full_fac
```

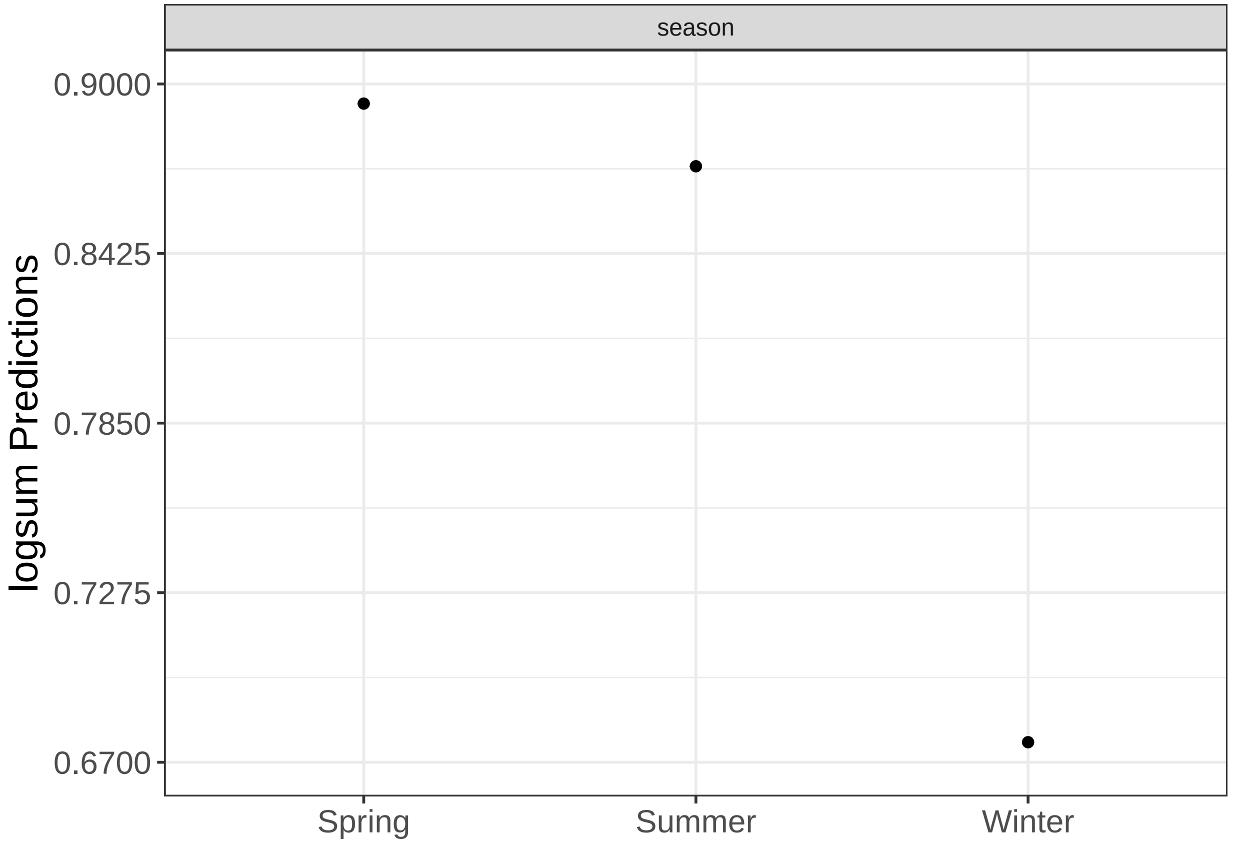
season
logsum Predictions
0.9000
0.8425
0.7850
0.7275
0.6700
Spring
Summer
Winter

Item 1: Tree Full vs RF Full

```
set.seed(123)
tree = rpart(logsum~., data = datafull)
rpart.plot(tree)
```

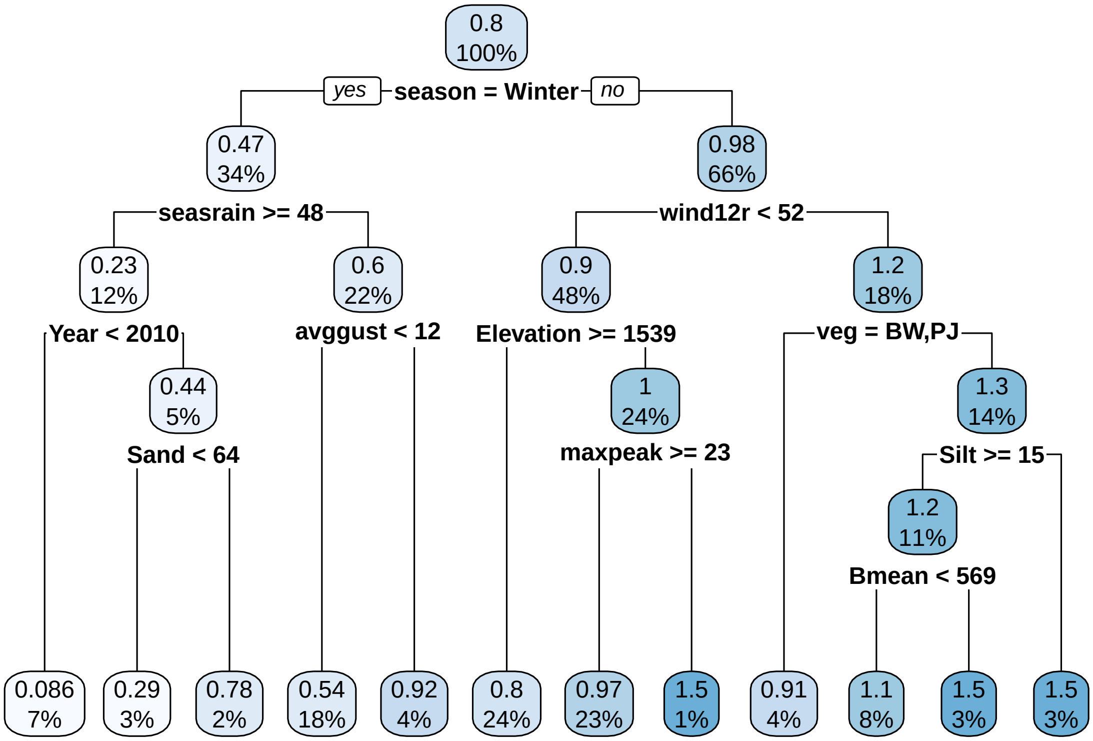


```
tree = rpart(logsum~., data = datafull,
             control = rpart.control(cp = 0.013, minsplit = 2))
plotcp(tree)
```

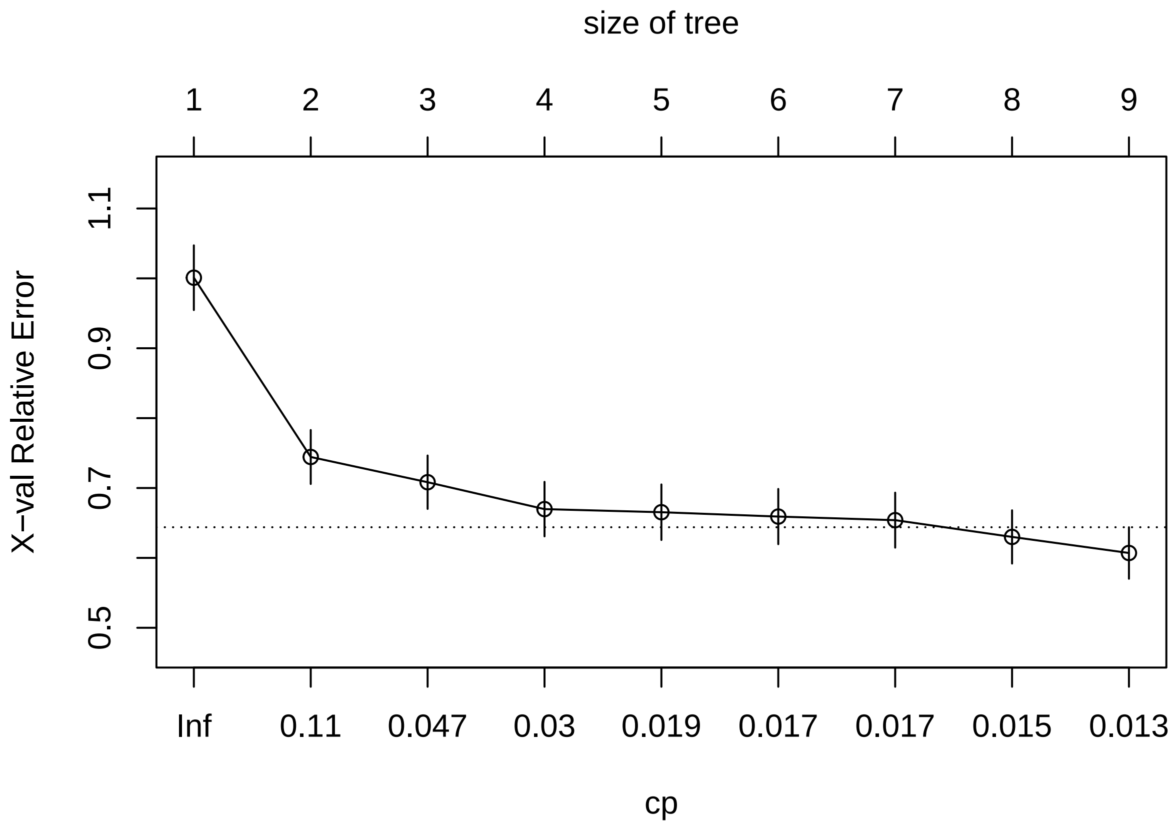


```r
rpart.plot(tree)
```

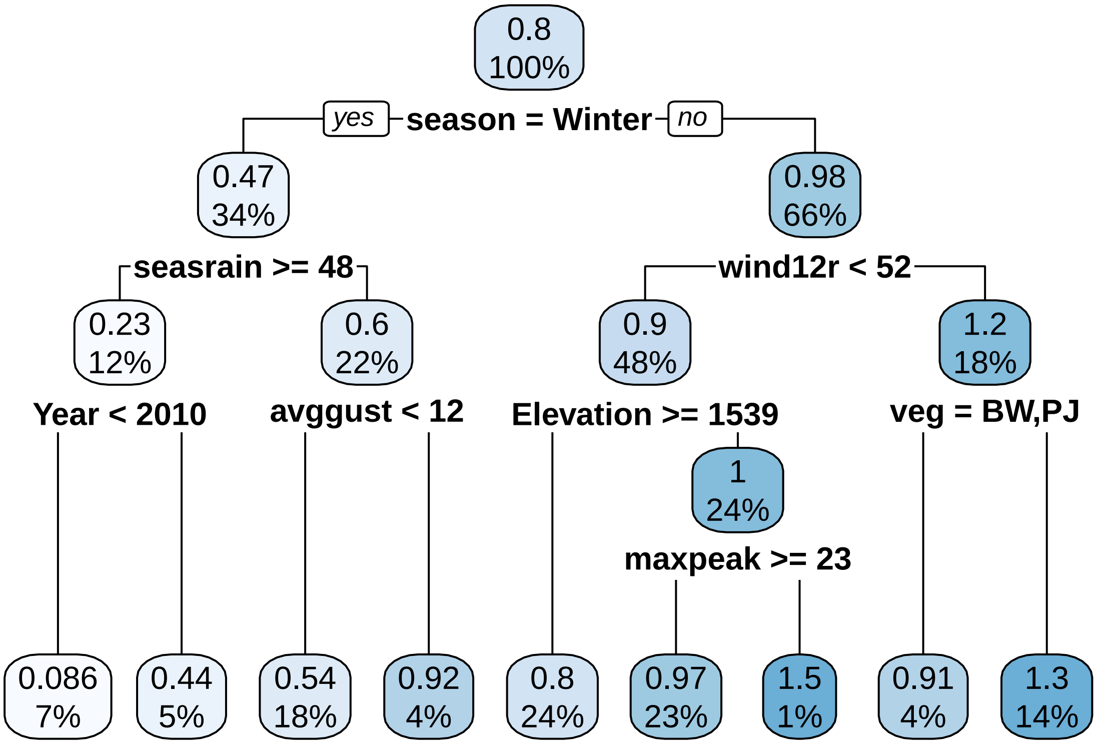


```r
set.seed(123)
fit_control <- caret::trainControl(method = "cv", number = 10)

xf <- caret::train(logsum~., method = "rpart", tuneLength = 25,
 trControl = fit_control, data = datafull)

xf$results[xf$results$cp == xf$bestTune$cp, c(1, 3)]
```

```
##             cp  Rsquared
## 11 0.006020502 0.4683415
```

```r
tree = rpart(logsum~., data = datafull,
             control = rpart.control(cp = xf$bestTune$cp, minsplit = 2))
rpart.plot(tree)
```

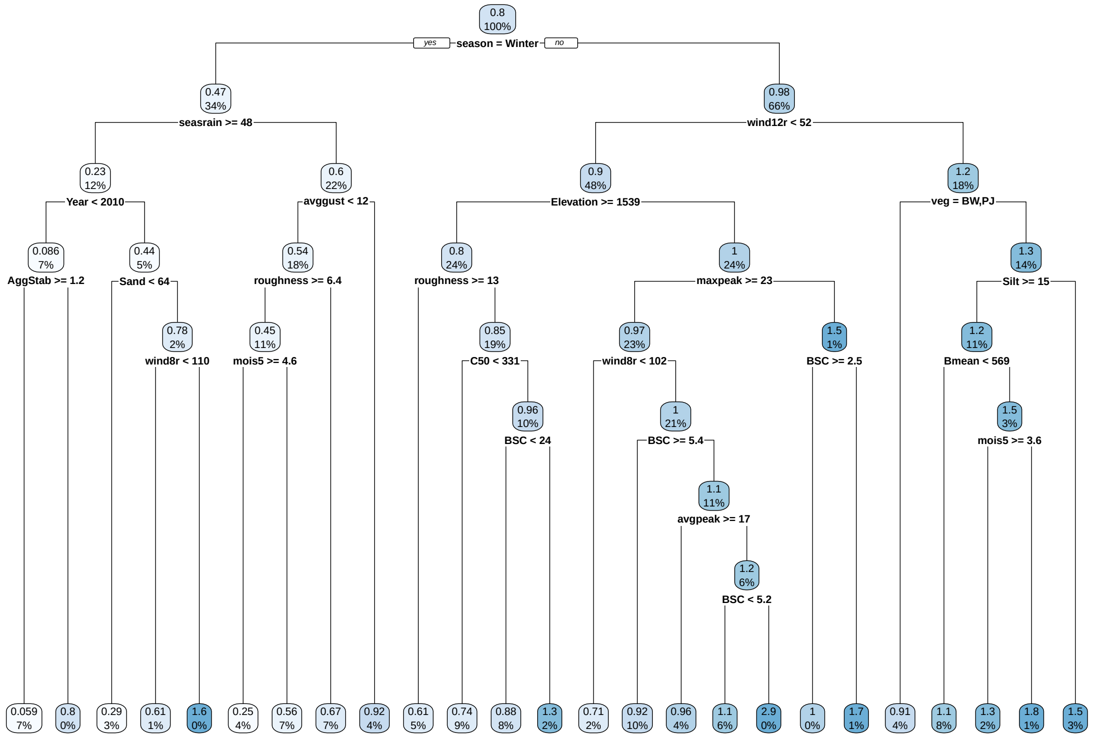


```r
tree$variable.importance
```

```
##        wind      season     wind12r      wind8r     maxpeak     avggust
## 101.5781255  88.3763266  55.2174404  52.7751762  51.2445032  42.4246829
##      wind4r     avgpeak        Year    seasrain         veg         BSC
##  32.5090448  25.0279658  22.2090262  21.0069971  14.7600283  11.0759589
##        C100   seasrainr       Bmean        Sand   roughness        B200
##  10.9204572  10.8178751   9.3555009   9.0893149   7.9875363   7.9317742
##   Elevation       Cmean        Silt         C50       mois5         C25
##   7.8995526   7.5543099   7.4900518   7.4882175   6.1455868   5.6728986
##      mois30      mois15     AggStab        C200   elev_type       Cover
##   5.6406183   5.4743566   5.4592555   5.3381045   4.3288268   3.4117781
##        B100        Clay     Annuals  BareGround         B50   condition
##   2.8239557   2.2093130   1.9213245   1.5290365   1.1057955   1.0172378
##         B25
##   0.3427573
```

```r
r2_fun(predict(tree, datafull), datafull$logsum)
```

```
## [1] 0.5753383
```

Item 2: RF pre15 vs RF Full

```
set.seed(123)

rf_pre15 <- randomForest(logsum~.,
                         data = data_pre15, importance = T)
rf_pre15$rsq[500]
```

```
## [1] 0.6208078
```

```
r2_fun(predict(rf_pre15, data_pre15), data_pre15$logsum)
```

```
## [1] 0.9171933
```

```
randomForestVIP::ggvip(rf_pre15, sqrt = F, scale = F, num_var = 10)$both_vips
```

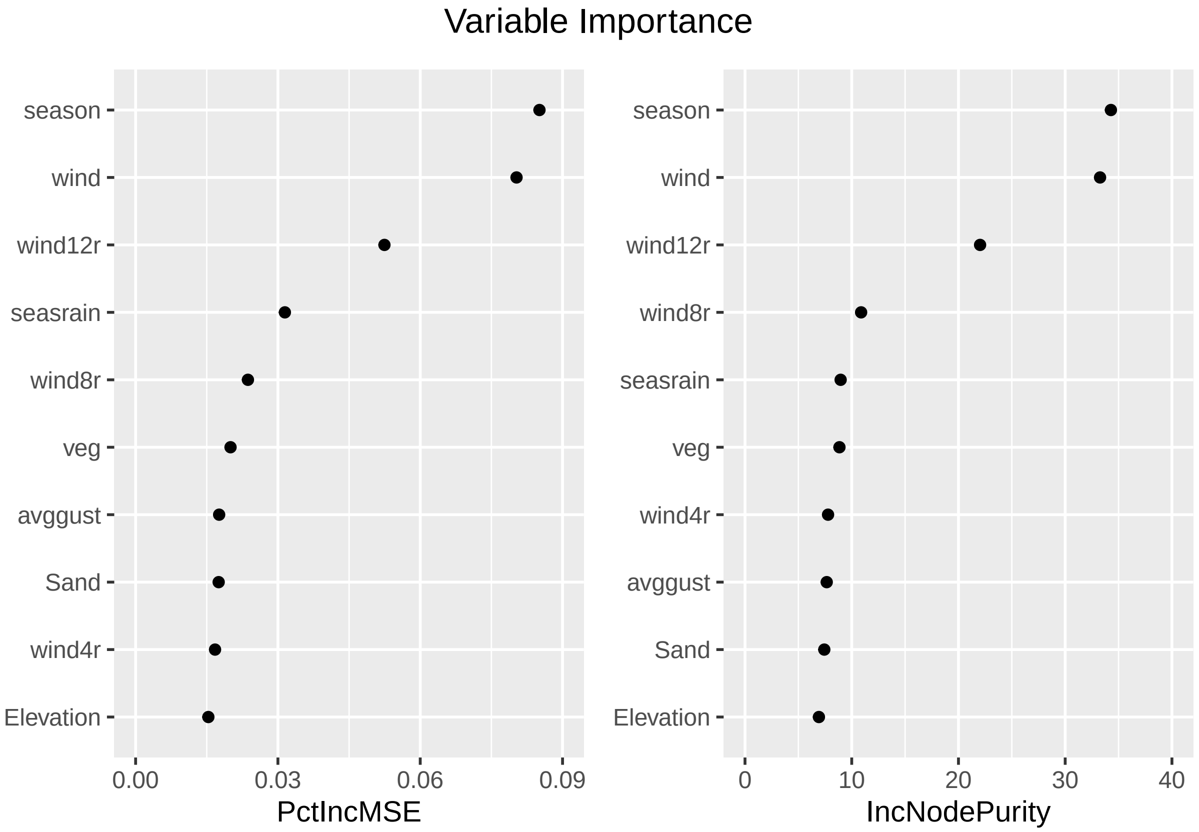


```
#ggsave("var_imp10.pdf", vg, dpi = 1600, width = 3, height = 4)

pd_res <- pdp_compare(rf_pre15, trellis = F,
                      var_vec = c("seasrain", "season",
                                  "wind", "avggust",
                                  "wind4r", "wind8r", "wind12r",
                                  "mois5", "mois15","mois30"))
pd_res$full_num
```

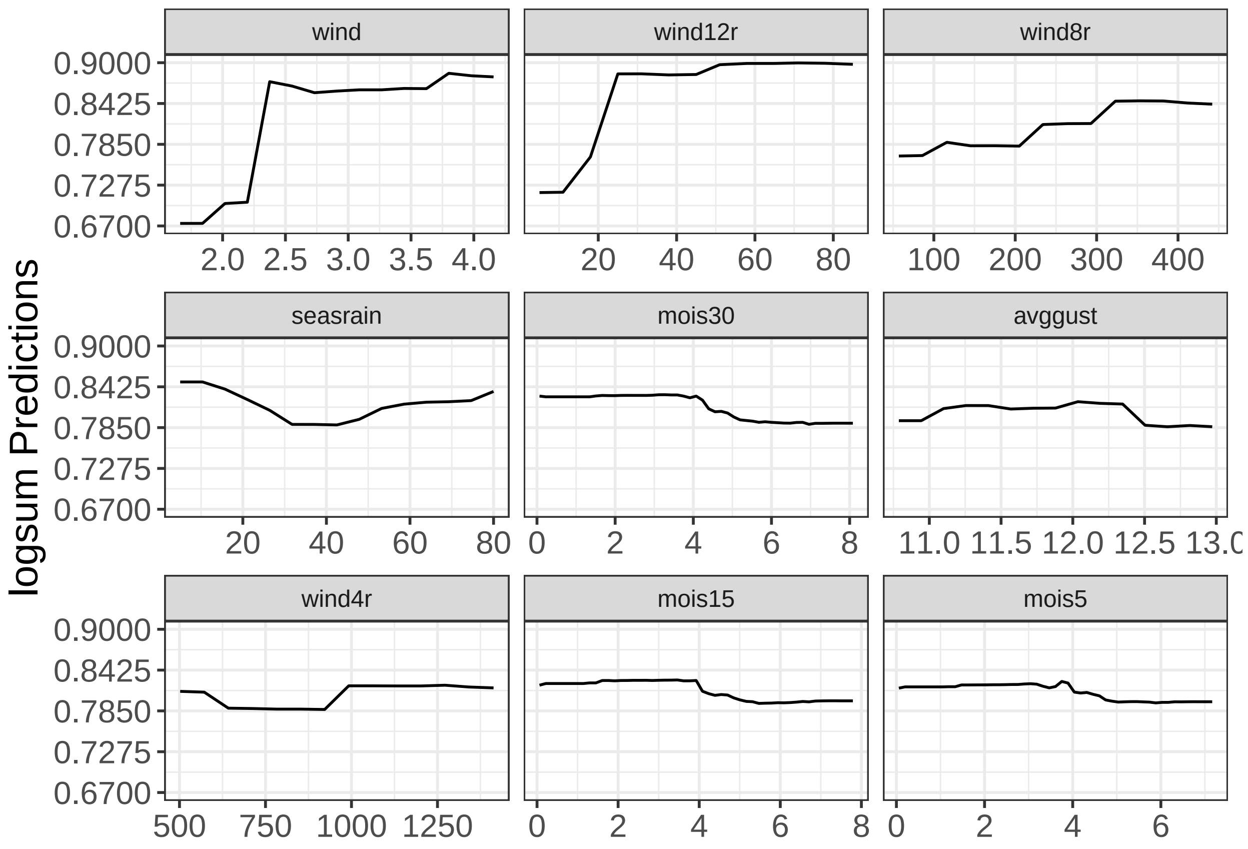


```
pd_res$full_fac
```

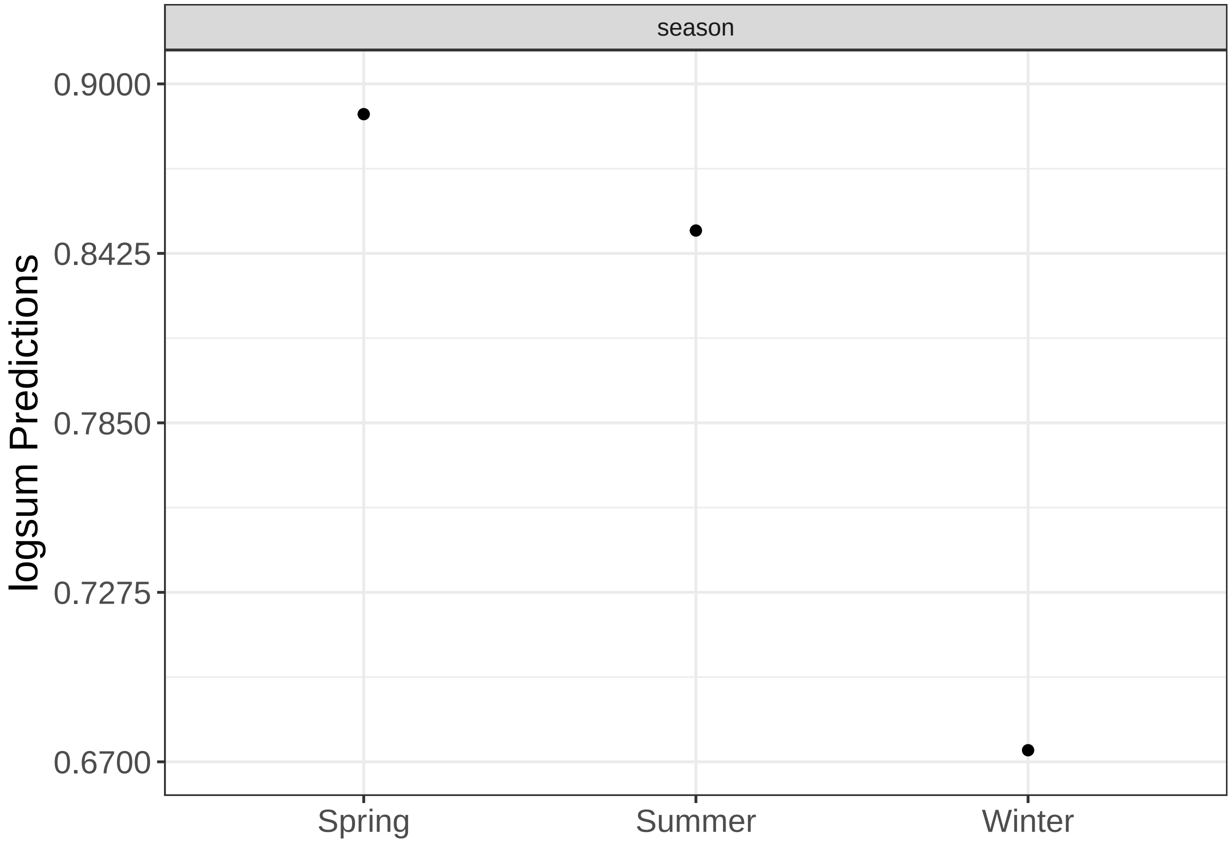
season
logsum Predictions
0.9000
0.8425
0.7850
0.7275
0.6700
Spring
Summer
Winter

Item 3: Moisture analysis

```
## Random Forest Full, No moisture
set.seed(123)
rf_nomois <- randomForest(logsum~.-mois5-mois15-mois30,
                          data = datafull, importance = T)
rf_nomois$rsq[500]
```

```
## [1] 0.6084891
```

```
r2_fun(predict(rf_nomois, datafull), datafull$logsum)
```

```
## [1] 0.9124028
```

```
randomForestVIP::ggvip(rf_nomois, sqrt = F, scale = F, num_var = 10)$both_vips
```

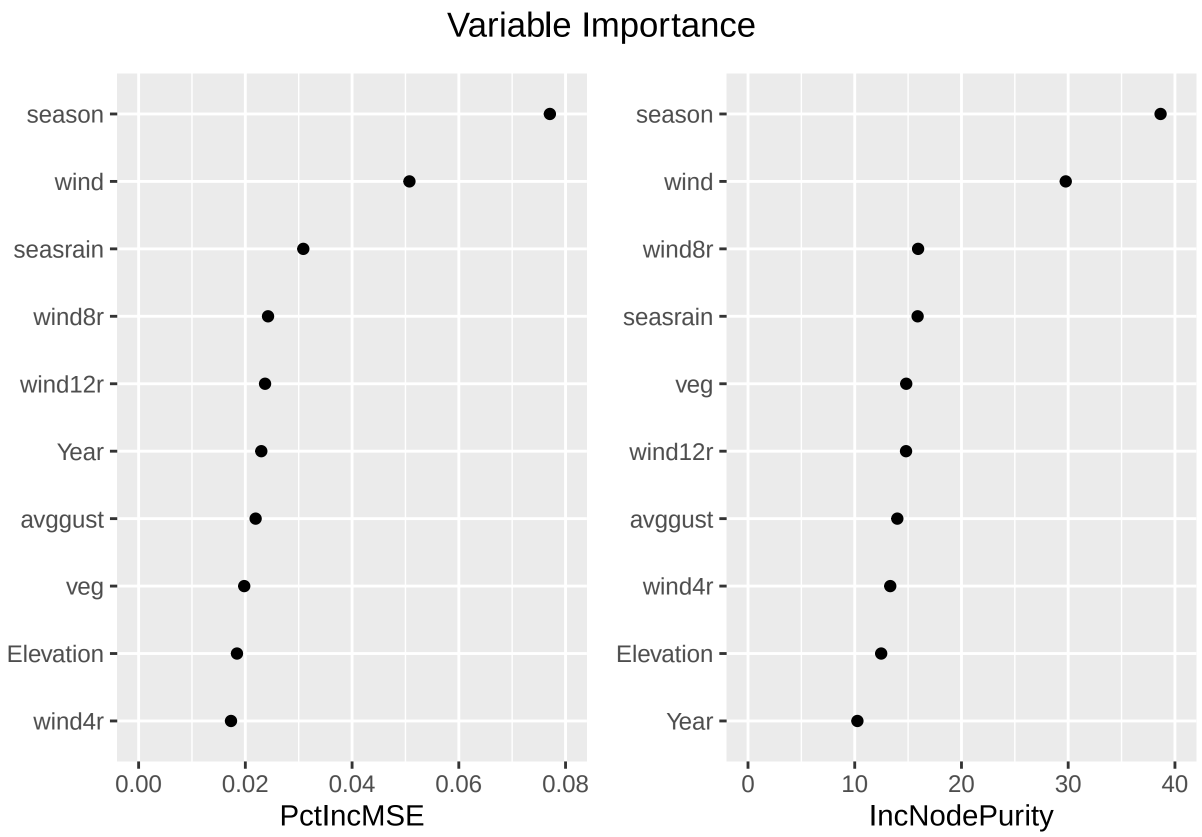


```
#ggsave("var_imp10.pdf", vg, dpi = 1600, width = 3, height = 4)

pd_res <- pdp_compare(rf_nomois, trellis = F,
                      var_vec = c("seasrain", "season",
                                  "wind", "avggust",
                                  "wind4r", "wind8r", "wind12r"))
pd_res$full_num
```

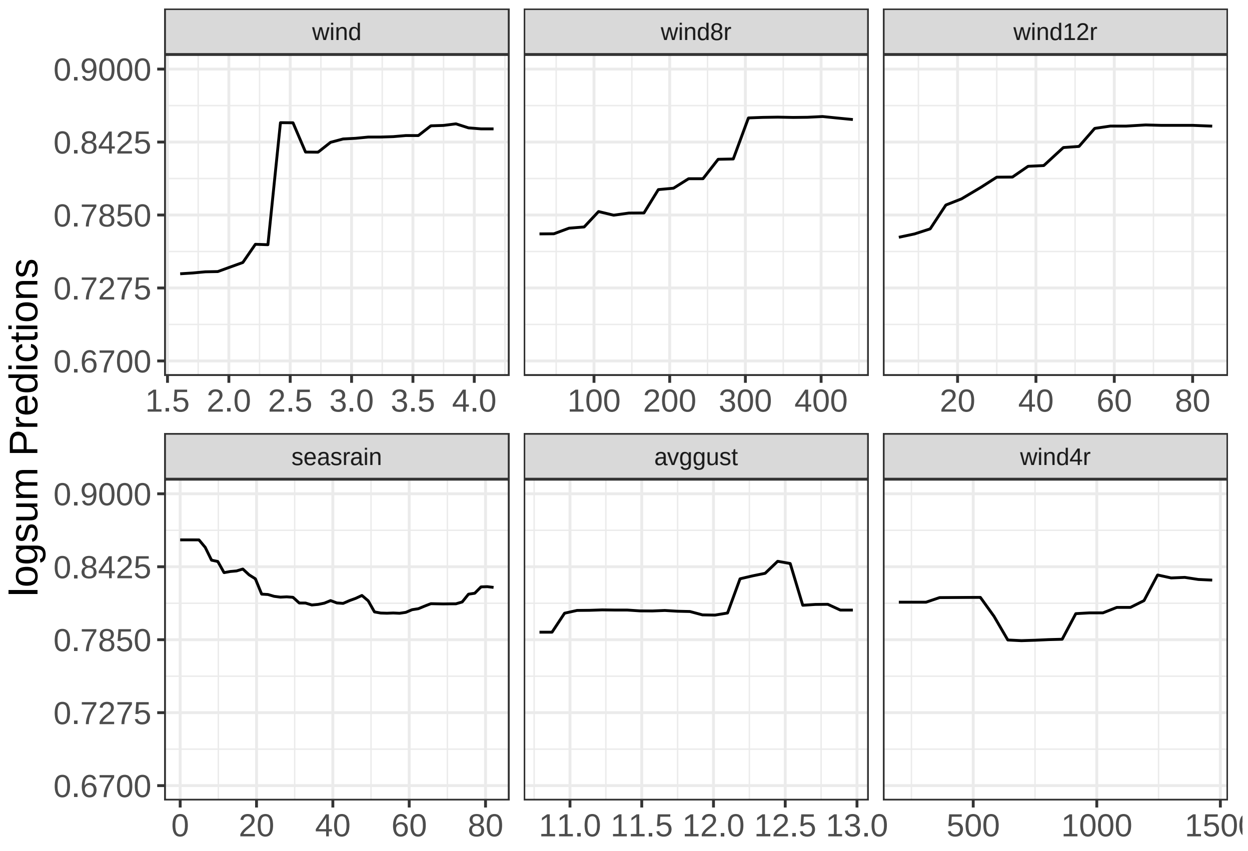


```
pd_res$full_fac
```

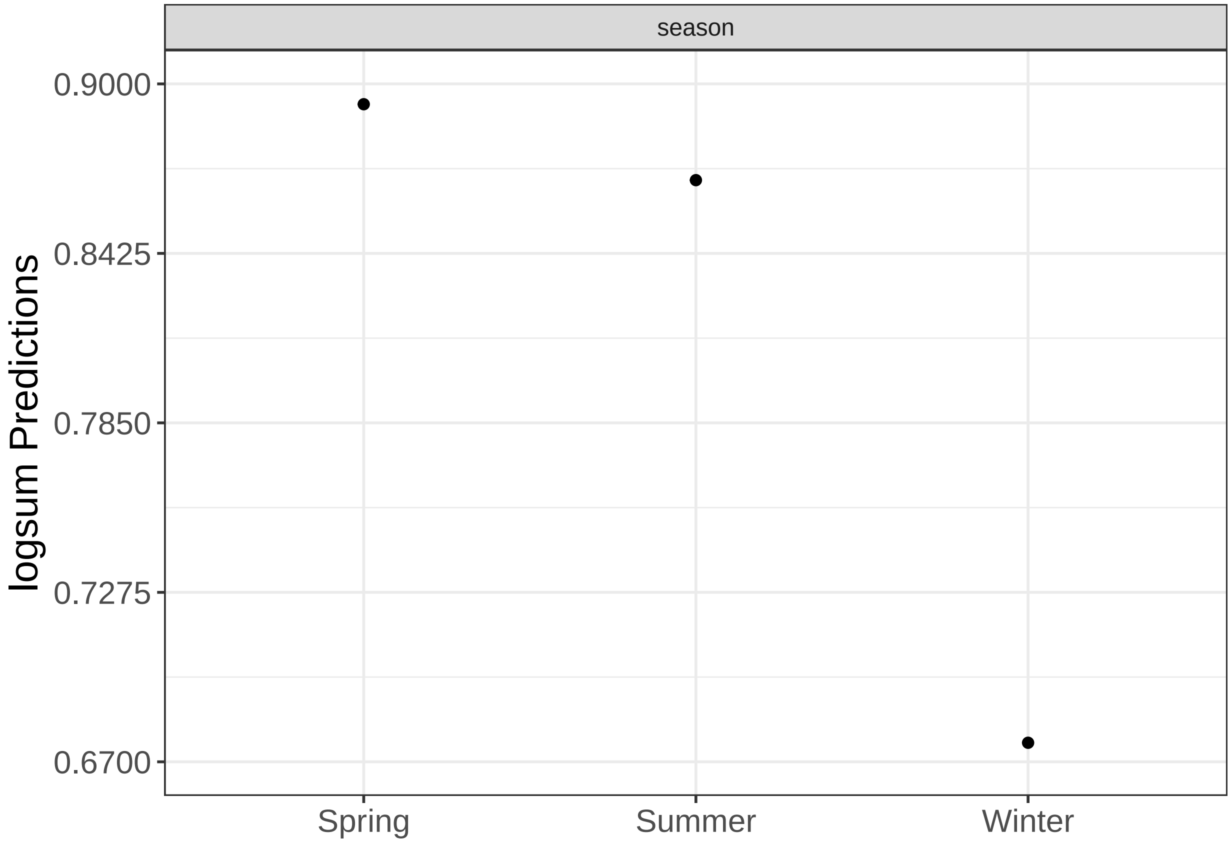


```r
## Random Forest Full, Only moisture
set.seed(123)
rf_mois <- randomForest(logsum~mois5+mois15+mois30,
                        data = datafull, importance = T)
rf_mois$rsq[500]
```

```
## [1] 0.3742136
```

```r
r2_fun(predict(rf_mois, datafull), datafull$logsum)
```

```
## [1] 0.5234519
```

```r
pd_res <- pdp_compare(rf_mois, trellis = F,
                      var_vec = c("mois5", "mois15","mois30"))
pd_res$full_num
```

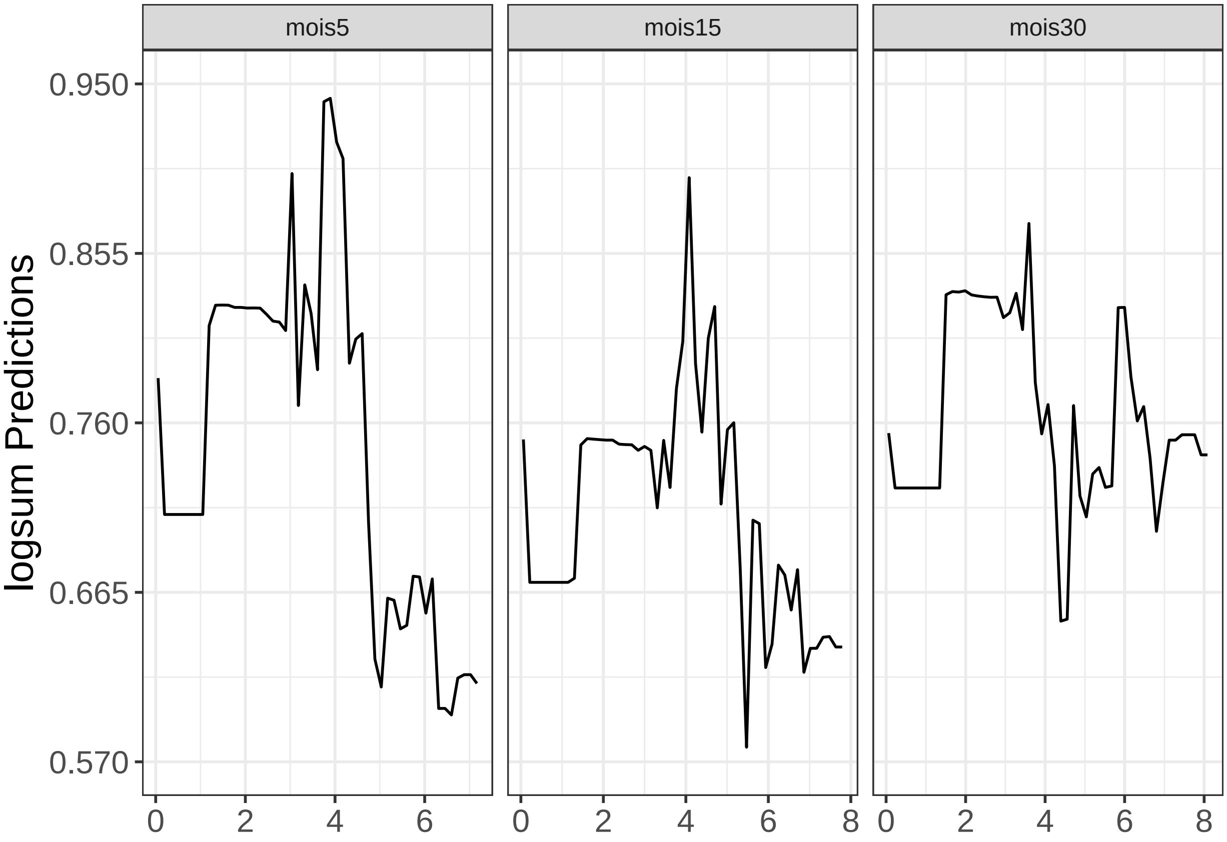


```
pd_res$full_fac
```

```
## NULL
```

Item 4: Graze and rough analysis

```
## Random Forest Full, no graze or rough
set.seed(123)
rf_nogr <- randomForest(logsum~.-condition-roughness,
                        data = datafull, importance = T)
rf_nogr$rsq[500]
```

```
## [1] 0.6086214
```

```
r2_fun(predict(rf_nogr, datafull), datafull$logsum)
```

```
## [1] 0.915737
```

```
randomForestVIP::ggvip(rf_nogr, sqrt = F, scale = F, num_var = 10)$both_vips
```

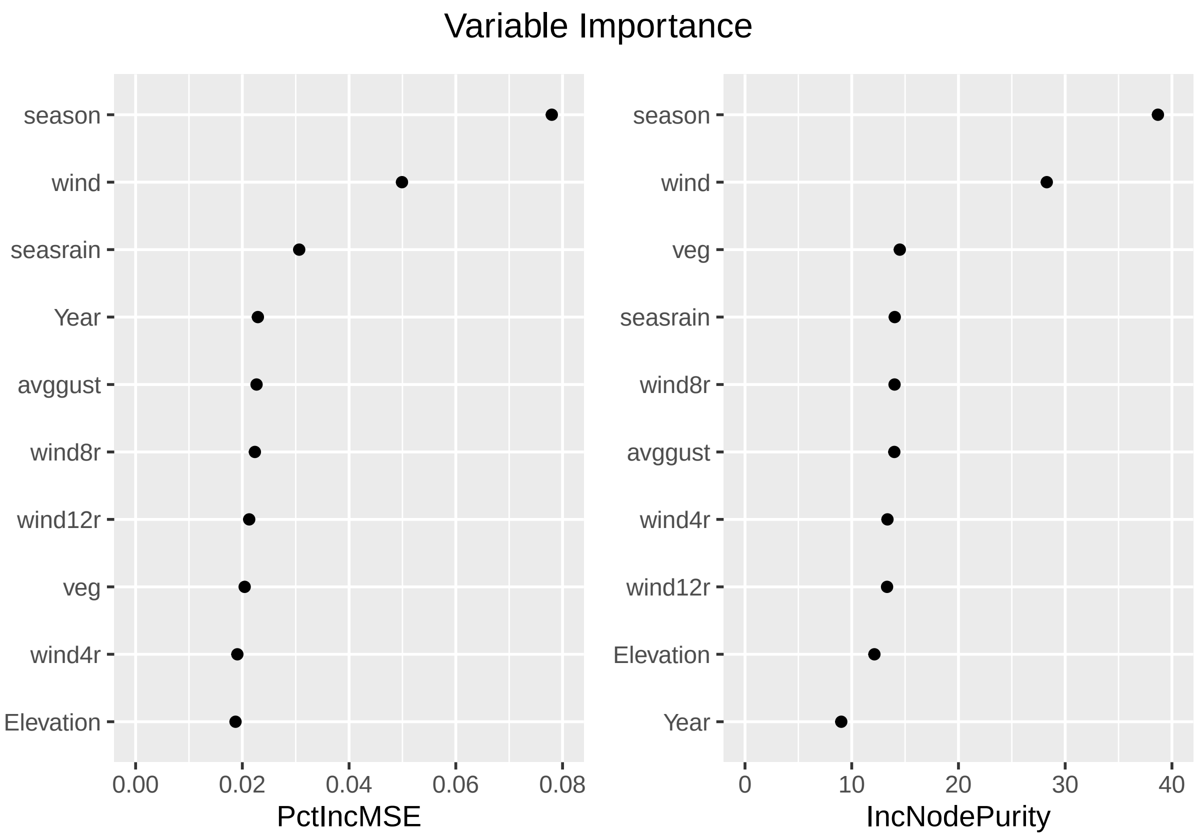


```
#ggsave("var_imp10.pdf", vg, dpi = 1600, width = 3, height = 4)

pd_res <- pdp_compare(rf_nogr, trellis = F,
                      var_vec = c("seasrain", "season",
                                  "wind", "avggust",
                                  "wind4r", "wind8r", "wind12r",
                                  "mois5", "mois15","mois30"))
pd_res$full_num
```

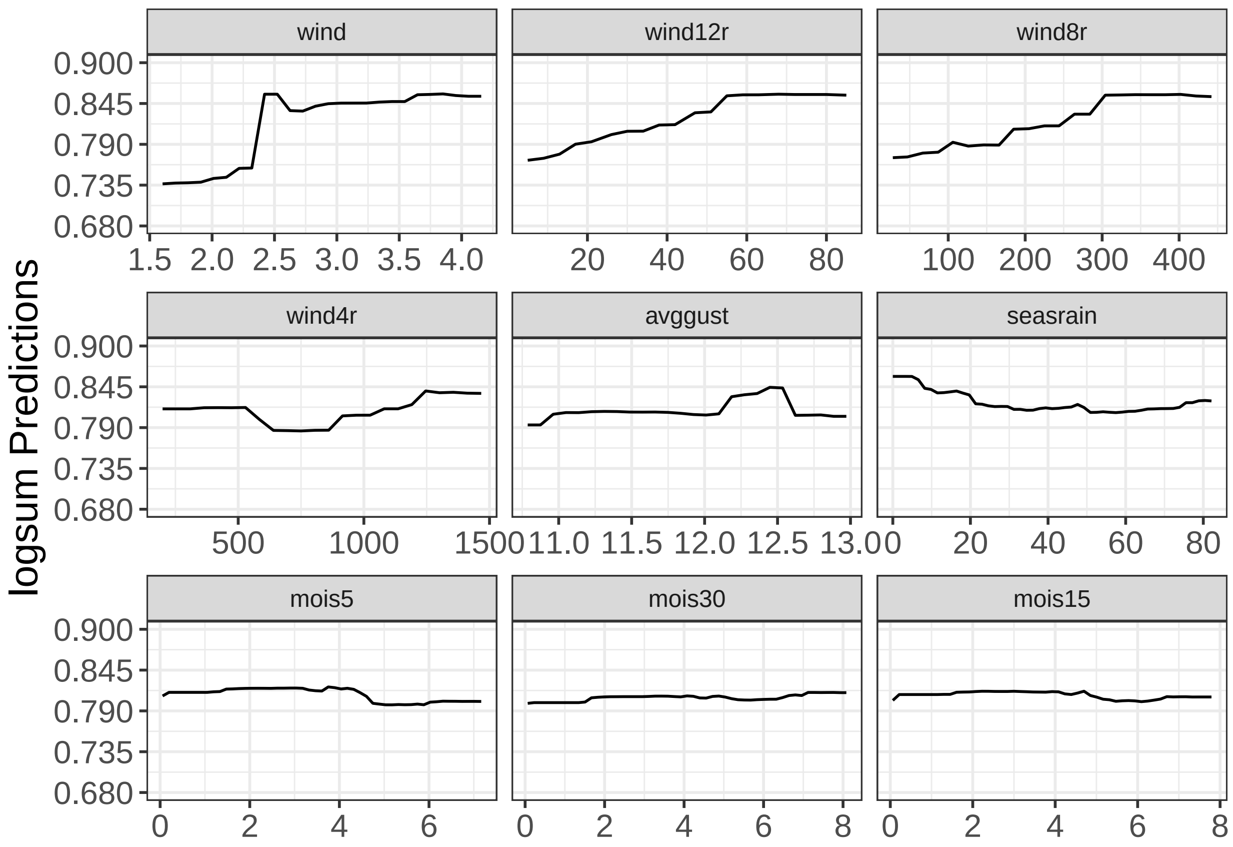


```
pd_res$full_fac
```

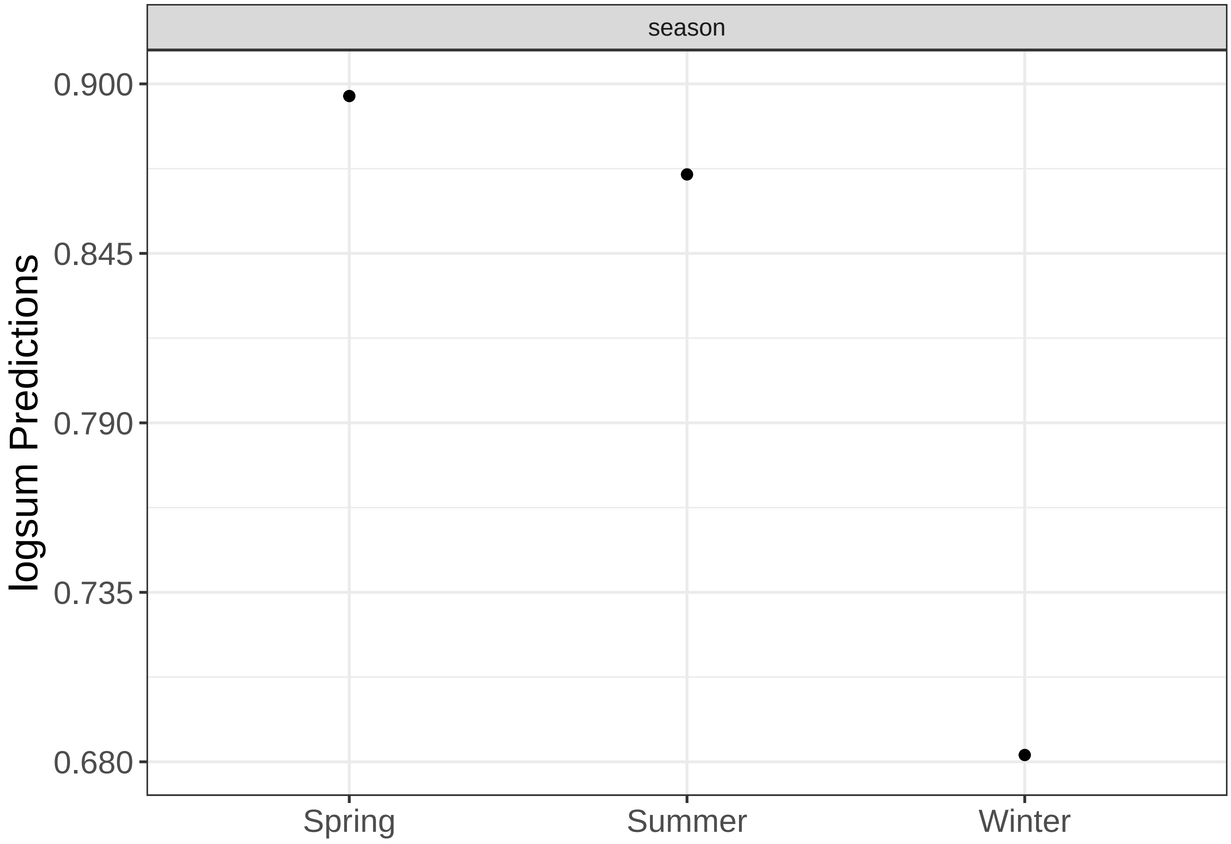


```
## Random Forest Full, Only graze and rough
set.seed(123)
rf_gr <- randomForest(logsum~condition+roughness,
                      data = datafull, importance = T)
rf_gr$rsq[500]
```

```
## [1] 0.06952724
```

```
r2_fun(predict(rf_gr, datafull), datafull$logsum)
```

```
## [1] 0.09354273
```

Item 5: Top Variables analysis

```
set.seed(123)
rf_t6 <- randomForest(logsum~season + wind + seasrain +
                        wind12r + wind8r + veg,
                        data = datafull, importance = T)
rf_t6$rsq[500]
```

```
## [1] 0.497245
```

```
r2_fun(predict(rf_t6, datafull), datafull$logsum)
```

```
## [1] 0.6191194
```

```
randomForestVIP::ggvip(rf_t6, sqrt = F, scale = F)$both_vips
```

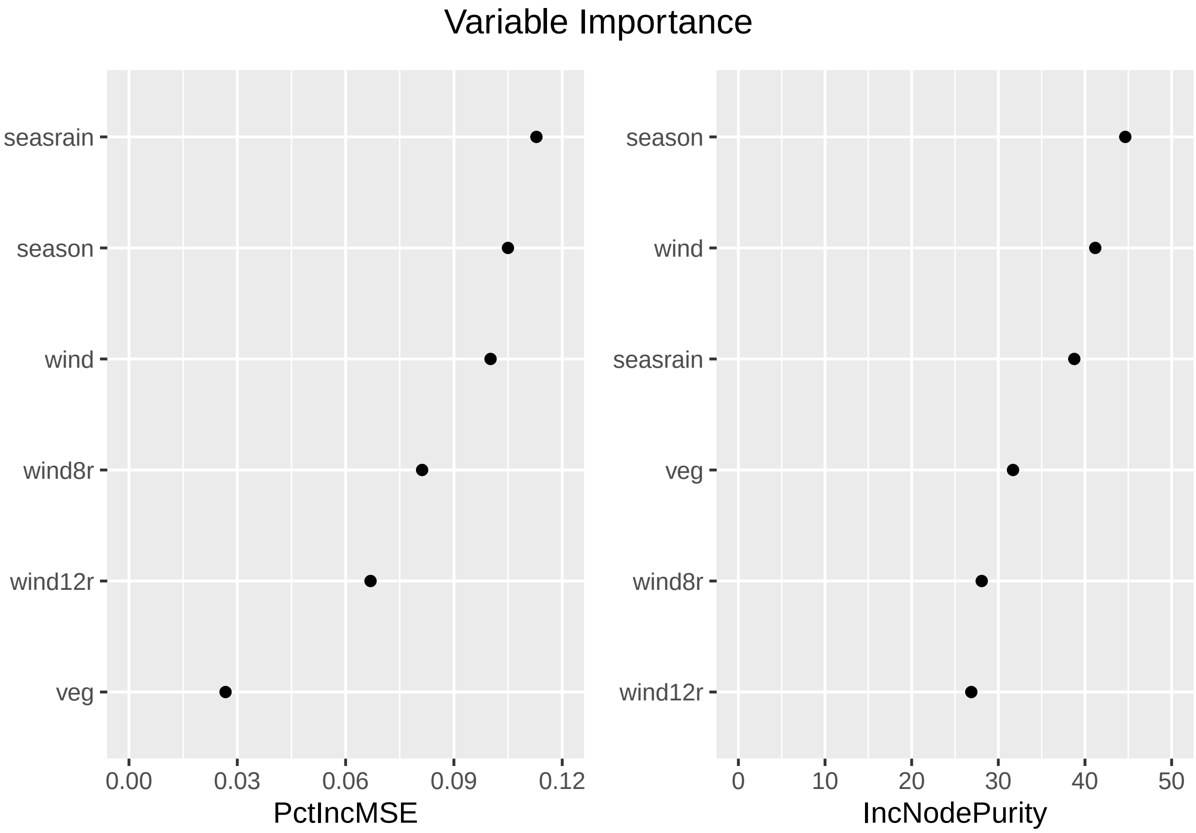


```
pd_res <- pdp_compare(rf_t6, trellis = F,
                      var_vec = c("seasrain", "season",
                                  "wind",
                                  "wind8r", "wind12r"))
pd_res$full_num
```

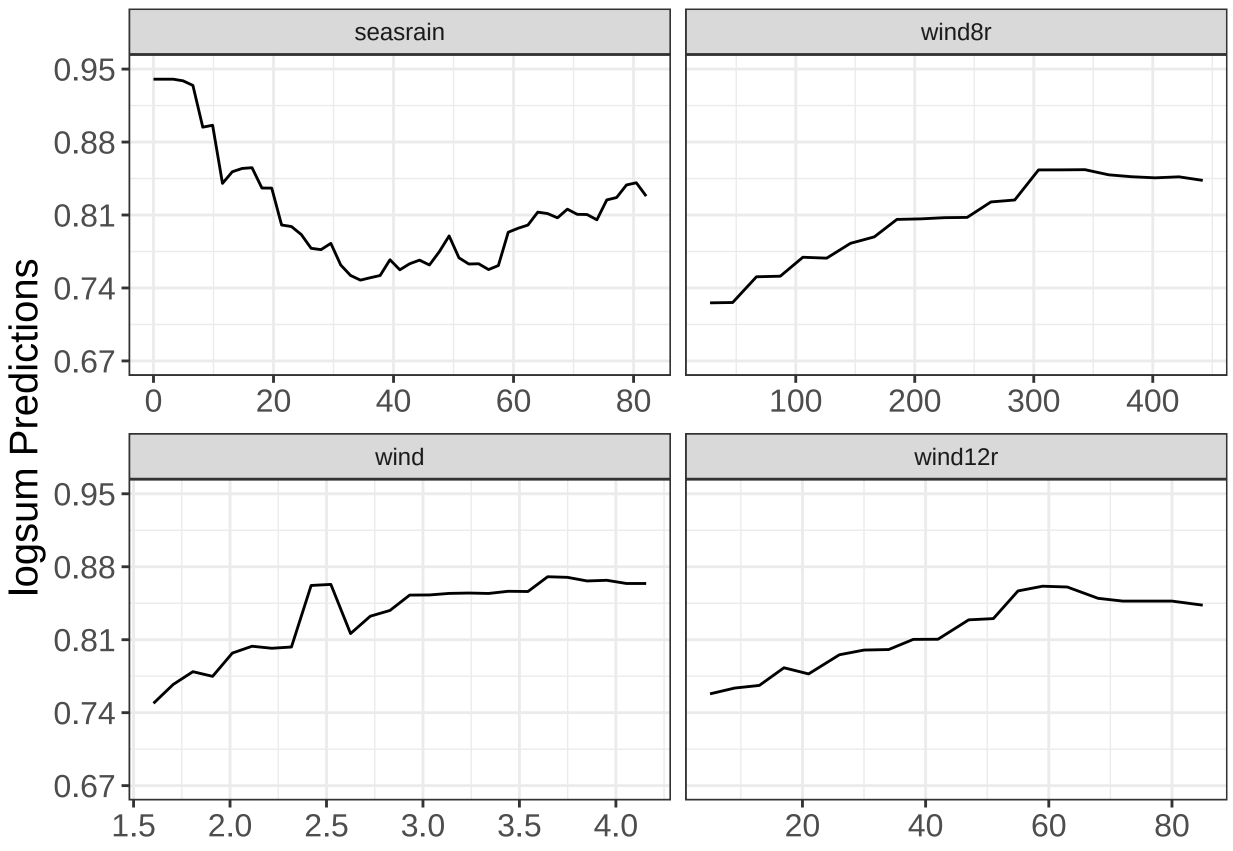


```
pd_res$full_fac
```

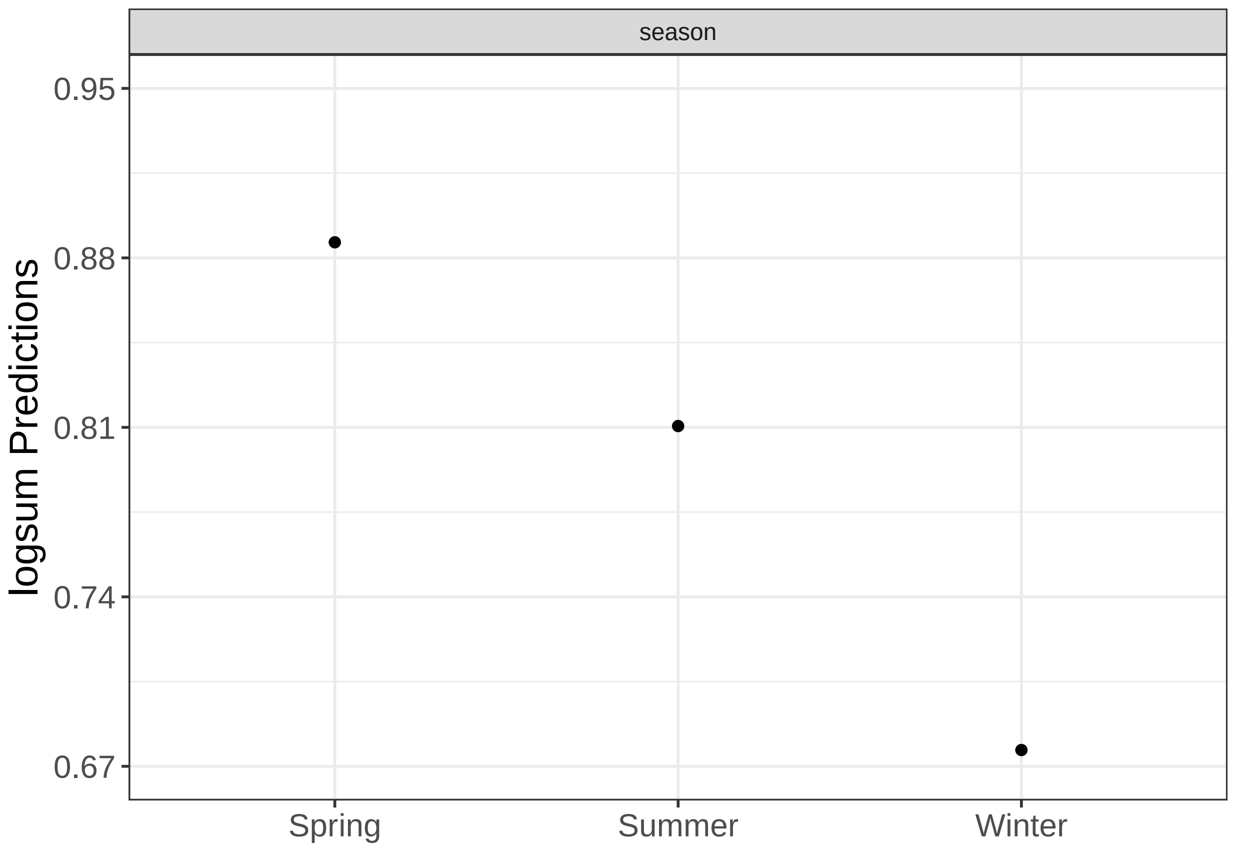

season
logsum Predictions
0.95
0.88
0.81
0.74
0.67
Spring
Summer
Winter

Item 6: Rerun full model Samples

```
set.seed(1)
data_s100 = datafull[sample(1:nrow(datafull), 100),]
r100 <- randomForest(logsum~., data = data_s100, importance = T)
r100$rsq[500]
```

```
## [1] 0.3314725
```

```
set.seed(12)
data_s300 = datafull[sample(1:nrow(datafull), 300),]
r300 <- randomForest(logsum~., data = data_s300, importance = T)
r300$rsq[500]
```

```
## [1] 0.5184938
```

```
set.seed(123)
data_s600 = datafull[sample(1:nrow(datafull), 600),]
r600 <- randomForest(logsum~., data = data_s600, importance = T)
r600$rsq[500]
```

```
## [1] 0.5583257
```

```
set.seed(1234)
data_s1200 = datafull[sample(1:nrow(datafull), 1200),]
r1200 <- randomForest(logsum~., data = data_s1200, importance = T)
r1200$rsq[500]
```

```
## [1] 0.6009019
```

Comparisons

Item 1: Model

```
train_R2 = c(tree_full = r2_fun(predict(tree, datafull), datafull$logsum),
       rf_full = r2_fun(predict(rf_full, datafull), datafull$logsum))

CV_R2 = c(tree_full = xf$results[xf$results$cp == xf$bestTune$cp, 3],
       rf_full = rf_full$rsq[500])
data.frame(train_R2 = round(train_R2, 3), CV_R2 = round(CV_R2, 3))
```

```
##             train_R2 CV_R2
## tree_full    0.575 0.468
## rf_full      0.917 0.610
```

Item 2: Data

```
train_R2 = c(rf_full = r2_fun(predict(rf_full, datafull), datafull$logsum),
       rf_pre15 = r2_fun(predict(rf_pre15, data_pre15), data_pre15$logsum))

CV_R2 = c(rf_full = rf_full$rsq[500],
       rf_pre15 = rf_pre15$rsq[500])
data.frame(train_R2 = round(train_R2, 3), CV_R2 = round(CV_R2, 3))
```

```
##            train_R2 CV_R2
## rf_full     0.917 0.610
## rf_pre15    0.917 0.621
```

Item 3: Moisture

```
train_R2 = c(rf_full = r2_fun(predict(rf_full, datafull), datafull$logsum),
             rf_no_mois = r2_fun(predict(rf_nomois, datafull), datafull$logsum),
             rf_only_mois = r2_fun(predict(rf_mois, datafull), datafull$logsum)
       )

CV_R2 = c(rf_full = rf_full$rsq[500],
       rf_no_mois = rf_nomois$rsq[500],
       rf_only_mois = rf_mois$rsq[500])
data.frame(train_R2 = round(train_R2, 3), CV_R2 = round(CV_R2, 3))
```

```
##                train_R2 CV_R2
## rf_full         0.917 0.610
## rf_no_mois      0.912 0.608
## rf_only_mois    0.523 0.374
```

Item 4: Graze and Roughness

```
train_R2 = c(rf_full = r2_fun(predict(rf_full, datafull), datafull$logsum),
             rf_no_gr = r2_fun(predict(rf_nogr, datafull), datafull$logsum),
             rf_gr = r2_fun(predict(rf_gr, datafull), datafull$logsum)
       )

CV_R2 = c(rf_full = rf_full$rsq[500],
       rf_no_gr = rf_nogr$rsq[500],
       rf_gr = rf_gr$rsq[500])
data.frame(train_R2 = round(train_R2, 3), CV_R2 = round(CV_R2, 3))
```

```
##          train_R2 CV_R2
## rf_full     0.917 0.610
## rf_no_gr    0.916 0.609
## rf_gr       0.094 0.070
```

Item 5: Top Variables

```
train_R2 = c(rf_full = r2_fun(predict(rf_full, datafull), datafull$logsum),
             rf_t6 = r2_fun(predict(rf_t6, datafull), datafull$logsum)
       )

CV_R2 = c(rf_full = rf_full$rsq[500],
       rf_t6 = rf_t6$rsq[500])
data.frame(train_R2 = round(train_R2, 3), CV_R2 = round(CV_R2, 3))
```

```
##         train_R2 CV_R2
## rf_full    0.917 0.610
## rf_t6      0.619 0.497
```

Item 6: Samples

```
train_R2 = c(rf_full = r2_fun(predict(rf_full, datafull), datafull$logsum),
             r100 = r2_fun(predict(r100, data_s100), data_s100$logsum),
             r300 = r2_fun(predict(r300, data_s300), data_s300$logsum),
             r600 = r2_fun(predict(r600, data_s600), data_s600$logsum),
             r1200 = r2_fun(predict(r1200, data_s1200), data_s1200$logsum)
       )

CV_R2 = c(rf_full = rf_full$rsq[500],
          r100 = r100$rsq[500],
          r300 = r300$rsq[500],
          r600 = r600$rsq[500],
          r1200 = r1200$rsq[500])
data.frame(train_R2 = round(train_R2, 3), CV_R2 = round(CV_R2, 3))
```

```
##          train_R2 CV_R2
## rf_full     0.917 0.610
## r100        0.874 0.331
## r300        0.911 0.518
## r600        0.911 0.558
## r1200       0.914 0.601
```

All R2 values except subsamples

```
train_R2 = c(rf_full = r2_fun(predict(rf_full, datafull), datafull$logsum),
       tree_full = r2_fun(predict(tree, datafull), datafull$logsum),
       rf_pre15 = r2_fun(predict(rf_pre15, data_pre15), data_pre15$logsum),
       rf_no_mois = r2_fun(predict(rf_nomois, datafull), datafull$logsum),
       rf_only_mois = r2_fun(predict(rf_mois, datafull), datafull$logsum),
       rf_no_gr = r2_fun(predict(rf_nogr, datafull), datafull$logsum),
       rf_gr = r2_fun(predict(rf_gr, datafull), datafull$logsum),
       rf_t6 = r2_fun(predict(rf_t6, datafull), datafull$logsum))

CV_R2 = c(rf_full = rf_full$rsq[500],
       tree_full = xf$results[xf$results$cp == xf$bestTune$cp, 3],
       rf_pre15 = rf_pre15$rsq[500],
       rf_no_mois = rf_nomois$rsq[500],
       rf_only_mois = rf_mois$rsq[500],
       rf_no_gr = rf_nogr$rsq[500],
       rf_gr = rf_gr$rsq[500],
       rf_t6 = rf_t6$rsq[500])
data.frame(train_R2 = round(train_R2, 3), CV_R2 = round(CV_R2, 3))
```

```
##               train_R2 CV_R2
## rf_full          0.917 0.610
## tree_full        0.575 0.468
## rf_pre15         0.917 0.621
## rf_no_mois       0.912 0.608
## rf_only_mois     0.523 0.374
## rf_no_gr         0.916 0.609
## rf_gr            0.094 0.070
## rf_t6            0.619 0.497
```

```
Sys.time() - s
```

```
## Time difference of 3.315536 mins
```
