# Supplementary material for: Season, wind speed, and seasonal rain are major drivers of a regional aeolian sediment transport model
Source: PLoS One. 2025 Sep 26;20(9):e0333166. doi: 10.1371/journal.pone.0333166 (PMC12468981; doi:10.1371/journal.pone.0333166)
Supplement: S2 — (DOCX) [file pone.0333166.s002.docx]

data dust;

set import;

run;

proc sort data=dust;

by season;

run;

data ldust;

set dust;

lflux=log(avgflux);

run;

proc glimmix data=ldust;

class season;

model lflux = season ;

lsmeans season / adjust=tukey;

run;

proc sort data=dust;

by condition;

run;

proc glimmix data=dust;

class condition;

model avgflux = condition;

lsmeans condition / adjust =tukey;

run;

proc sort data=ldust;

by veg;

run;

data vegtight;

set ldust;

if veg = 'BW' then delete;

if veg = 'MC' then delete;

if veg = 'MB' then delete;

;

run;

proc glimmix data=vegtight;

class veg;

model lflux = veg ;

lsmeans veg / adjust=tukey;

run;
