# Supplementary material for: Season, wind speed, and seasonal rain are major drivers of a regional aeolian sediment transport model
Source: PLoS One. 2025 Sep 26;20(9):e0333166. doi: 10.1371/journal.pone.0333166 (PMC12468981; doi:10.1371/journal.pone.0333166)
Supplement: Attachment — Submitted filename: response to reviewers.pdf [file pone.0333166.s003.pdf]

Response to reviewer comments

Please find point-by-point responses below.

PONE-D-25-20429

Season, wind speed, and seasonal rain are major drivers of a regional sediment transport model

PLOS ONE

Dear Dr. Kulmatiski,

Thank you for submitting your manuscript to PLOS ONE. After careful consideration, we feel that it has merit but does not fully meet PLOS ONE's publication criteria as it currently stands. Therefore, we invite you to submit a revised version of the manuscript that addresses the points raised during the review process.

==============================

Dear Authors,

We have received two reviews of your manuscript, both highlighting minor revisions to be made. The primary concern raised by the reviewers is the need for more detailed explanations in the Methods section.

Additionally, we encourage you to strengthen your argument in the Discussion and Introduction sections, particularly in emphasizing the novelty of your contribution.

Please ensure that all necessary corrections requested by the reviewers are incorporated into your revised manuscript.

We look forward to receiving your updated submission.

Sincerely,
Academic Editor

---

Thanks for this opportunity to revise our manuscript. You will find point-by-point responses below. As suggested, we have also strengthened the Introduction and Discussion by

highlighting the novelty of this paper.

Please submit your revised manuscript by Aug 05 2025 11:59PM. If you will need more time than this to complete your revisions, please reply to this message or contact the journal office at plosone@plos.org. When you're ready to submit your revision, log on to https://www.editorialmanager.com/pone/ and select the 'Submissions Needing Revision' folder to locate your manuscript file.

Please include the following items when submitting your revised manuscript:

- A rebuttal letter that responds to each point raised by the academic editor and reviewer(s). You should upload this letter as a separate file labeled 'Response to Reviewers'.
- A marked-up copy of your manuscript that highlights changes made to the original version. You should upload this as a separate file labeled 'Revised Manuscript with Track Changes'.
- An unmarked version of your revised paper without tracked changes. You should upload this as a separate file labeled 'Manuscript'.

If you would like to make changes to your financial disclosure, please include your updated statement in your cover letter. Guidelines for resubmitting your figure files are available below the reviewer comments at the end of this letter.

If applicable, we recommend that you deposit your laboratory protocols in protocols.io to enhance the reproducibility of your results. Protocols.io assigns your protocol its own identifier (DOI) so that it can be cited independently in the future. For instructions see: https://journals.plos.org/plosone/s/submission-guidelines#loc-laboratory-protocols. Additionally, PLOS ONE offers an option for publishing peer-reviewed Lab Protocol articles, which describe protocols hosted on protocols.io. Read more information on sharing protocols at https://plos.org/protocols?utm_medium=editorial-email&utm_source=authorletters&utm_campaign=protocols.

We look forward to receiving your revised manuscript.

Kind regards,

Jamil Alexandre Ayach Anache
Academic Editor
PLOS ONE

**Journal Requirements:**

1. When submitting your revision, we need you to address these additional requirements.

Please ensure that your manuscript meets PLOS ONE's style requirements, including those for file naming. The PLOS ONE style templates can be found at

https://journals.plos.org/plosone/s/file?id=wjVg/PLOSOne_formatting_sample_main_body.pdf and

https://journals.plos.org/plosone/s/file?id=ba62/PLOSOne_formatting_sample_title_authors_affiliations.pdf

Response: Thanks, we have reformatted the manuscript to meet PLOS ONE's style requirements.

2. In your Methods section, please provide additional information regarding the permits you obtained for the work. Please ensure you have included the full name of the authority that approved the field site access and, if no permits were required, a brief statement explaining why.

Response: We have added the following to the methods: 'This study involved collecting dust samples from an established network of dust collectors placed on public lands. No permits or sampling ethics approvals were needed for this work.'

3. Please note that PLOS ONE has specific guidelines on code sharing for submissions in which author-generated code underpins the findings in the manuscript. In these cases, we expect all author-generated code to be made available without restrictions upon publication of the work. Please review our guidelines at https://journals.plos.org/plosone/s/materials-and-software-sharing#loc-sharing-code and ensure that your code is shared in a way that follows best practice and facilitates reproducibility and reuse.

Response: We have included our code as appendices in the revision.

4. Thank you for stating in your Funding Statement:

This research was supported by the Utah Agricultural Experiment Station, Utah State University, and approved as journal paper number 9854. The Ministry of National Education and the Ministry of Agriculture and Forest of Turkey supported M. Ozturk.

Please provide an amended statement that declares *all* the funding or sources of support (whether external or internal to your organization) received during this study, as detailed online in our guide for authors at http://journals.plos.org/plosone/s/submit-now. Please also include the statement "There was no additional external funding received for this study." in your updated Funding Statement.

Please include your amended Funding Statement within your cover letter. We will change the online submission form on your behalf.

Response: We have amended our Funding Statement as suggested and attached the revised statement to our cover letter.

5. We noted in your submission details that a portion of your manuscript may have been presented or published elsewhere. "Datasets used in the research were published separately, but not analyzed. Tyree, G.L., L. A. Zeller, J. Belnap, E. L. Geiger, T. W. Nauman, and M. C. Duniway. 2024, "Dust mass and horizontal aeolian sediment flux data from a sampler network on the Colorado Plateau, USA." U.S. Geological Survey data release. https://doi.org/10.5066/P1ZHQX9W".

Please clarify whether this [conference proceeding or publication] was peer-reviewed and formally published. If this work was previously peer-reviewed and published, in the cover letter please provide the reason that this work does not constitute dual publication and should be included in the current manuscript.

Response: The dataset was published by the US Geological Survey, but was not peer-reviewed or analyzed. Simply the raw data was published. We have addressed this point in our cover letter.

6. Please include your full ethics statement in the 'Methods' section of your manuscript file. In your statement, please include the full name of the IRB or ethics committee who approved or waived your study, as well as whether or not you obtained informed written or verbal consent. If consent was waived for your study, please include this information in your statement as well.

Response: We have added the following to the Methods: 'This study involved collecting dust samples from an established network of dust collectors placed on public lands. No permits or sampling ethics approvals were needed for this work.'

7. We note that Figure 1 in your submission contain satellite images which may be copyrighted. All PLOS content is published under the Creative Commons Attribution License (CC BY 4.0), which means that the manuscript, images, and Supporting Information files will be freely available online, and any third party is permitted to access, download, copy, distribute, and use these materials in any way, even commercially, with proper attribution. For these reasons, we cannot publish previously copyrighted maps or satellite images created using proprietary data, such as Google software (Google Maps, Street View, and Earth). For more information, see our copyright guidelines: http://journals.plos.org/plosone/s/licenses-and-copyright.

We require you to either present written permission from the copyright holder to publish these figures specifically under the CC BY 4.0 license, or remove the figures from your submission:

a. You may seek permission from the original copyright holder of Figure 1 to publish the content specifically under the CC BY 4.0 license.

We recommend that you contact the original copyright holder with the Content Permission Form (http://journals.plos.org/plosone/s/file?id=7c09/content-permission-form.pdf) and the following text:

“I request permission for the open-access journal PLOS ONE to publish XXX under the Creative Commons Attribution License (CCAL) CC BY 4.0 (http://creativecommons.org/licenses/by/4.0/). Please be aware that this license allows unrestricted use and distribution, even commercially, by third parties. Please reply and provide explicit written permission to publish XXX under a CC BY license and complete the attached form.”

Please upload the completed Content Permission Form or other proof of granted permissions as an "Other" file with your submission.

In the figure caption of the copyrighted figure, please include the following text: “Reprinted from [ref] under a CC BY license, with permission from [name of publisher], original copyright [original copyright year].”

b. If you are unable to obtain permission from the original copyright holder to publish these figures under the CC BY 4.0 license or if the copyright holder’s requirements are

incompatible with the CC BY 4.0 license, please either i) remove the figure or ii) supply a replacement figure that complies with the CC BY 4.0 license. Please check copyright information on all replacement figures and update the figure caption with source information. If applicable, please specify in the figure caption text when a figure is similar but not identical to the original image and is therefore for illustrative purposes only.

The following resources for replacing copyrighted map figures may be helpful:

USGS National Map Viewer (public domain): http://viewer.nationalmap.gov/viewer/

The Gateway to Astronaut Photography of Earth (public domain): http://eol.jsc.nasa.gov/sseop/clickmap/

Maps at the CIA (public domain): https://www.cia.gov/library/publications/the-world-factbook/index.html and https://www.cia.gov/library/publications/cia-maps-publications/index.html

NASA Earth Observatory (public domain): http://earthobservatory.nasa.gov/

Landsat: http://landsat.visibleearth.nasa.gov/

USGS EROS (Earth Resources Observatory and Science (EROS) Center) (public domain): http://eros.usgs.gov/#

Natural Earth (public domain): http://www.naturalearthdata.com/

Response: We have changed the figure and now include a publicly available USGS map.

8. Please review your reference list to ensure that it is complete and correct. If you have cited papers that have been retracted, please include the rationale for doing so in the manuscript text, or remove these references and replace them with relevant current references. Any changes to the reference list should be mentioned in the rebuttal letter that accompanies your revised manuscript. If you need to cite a retracted article, indicate the article's retracted status in the References list and also include a citation and full reference for the retraction notice.

Response: We have reformatted the References to meet Plos One style requirements. We are aware of no papers that have been retracted.

**Additional Editor Comments:**

Dear Authors,
We have received two reviews of your manuscript, both highlighting minor revisions to be made. The primary concern raised by the reviewers is the need for more detailed explanations in the Methods section.
Additionally, we encourage you to strengthen your argument in the Discussion and Introduction sections, particularly in emphasizing the novelty of your contribution.
Please ensure that all necessary corrections requested by the reviewers are incorporated into your revised manuscript.
We look forward to receiving your updated submission.
Sincerely,
Academic Editor

Response: Thanks for this opportunity to revise our manuscript. We have added the following comments to the Introduction and Discussion to clarify the novel contribution of this manuscript:

In the Introduction we have added the following: 'Soil moisture, therefore, is known to be important at small scales and is likely to be important at large scales, but we are not aware of a study that has assessed the effect of soil moisture on sediment transport at large scales. Here, we address this knowledge gap by including estimates of soil moisture as a predictor of sediment flux in regional sediment flux measurement network.'

In the Discussion, we have added the following:' This study improved upon previous analyses of a regional dust flux dataset by 1) testing the effects of soil moisture, 2) greater dataset size, 3) soil roughness measurements, and by using a random forest (RF) modeling approach, which was previously found to improve upon previous regression tree modeling approaches.'

[Note: HTML markup is below. Please do not edit.]

Reviewers' comments:

Reviewer's Responses to Questions

**Comments to the Author**

1. Is the manuscript technically sound, and do the data support the conclusions?

The manuscript must describe a technically sound piece of scientific research with data that supports the conclusions. Experiments must have been conducted rigorously, with appropriate controls, replication, and sample sizes. The conclusions must be drawn appropriately based on the data presented.

Reviewer #1: Yes

Reviewer #2: Yes

---

2. Has the statistical analysis been performed appropriately and rigorously?

Reviewer #1: Yes

Reviewer #2: Yes

---

3. Have the authors made all data underlying the findings in their manuscript fully available?

The PLOS Data policy requires authors to make all data underlying the findings described in their manuscript fully available without restriction, with rare exception (please refer to the Data Availability Statement in the manuscript PDF file). The data should be provided as part of the manuscript or its supporting information, or deposited to a public repository. For example, in addition to summary statistics, the data points behind means, medians and variance measures should be available. If there are restrictions on publicly sharing data—e.g. participant privacy or use of data from a third party—those must be specified.

Reviewer #1: Yes

Reviewer #2: Yes

---

4. Is the manuscript presented in an intelligible fashion and written in standard English?

PLOS ONE does not copyedit accepted manuscripts, so the language in submitted articles

must be clear, correct, and unambiguous. Any typographical or grammatical errors should be corrected at revision, so please note any specific errors here.

Reviewer #1: Yes

Reviewer #2: Yes

---

5. Review Comments to the Author

Please use the space provided to explain your answers to the questions above. You may also include additional comments for the author, including concerns about dual publication, research ethics, or publication ethics. (Please upload your review as an attachment if it exceeds 20,000 characters)

**Reviewer #1:** Summary: in this study, the authors tested several statistical models to explain the variance observed in a dataset of aeolian (horizontal) sediment fluxes measured from 2004 to 2018 in a network of 52 masts of sand traps located on the Colorado Plateau (USA). They showed that the use of a random forest statistical model instead of a regression tree allowed explaining 91% of the variance in sediment transport vs. 58% before. Increasing sample size (adding 4 years of measurements) did not improve the results so does adding information on soil moisture, grazing condition or soil roughness. The random forest model developed by the authors allow classifying the parameters that explain the variance observed in the dataset with wind speed being the primary determinant of sediment transport, followed by soil moisture.

Major comment:
The study proposed by Kulmatiski et al. is of interest because it discusses the interest of using random forest statistical models to explain the variance observed in large dataset and gives insight on how to optimize its use. It also gives advice on the requirements of future networks to analyse aeolian sediment transport at landscape scale. The manuscript is well organized and written. All findings are supported by the results presented in the study. However, some clarifications in the Materials and Methods section are needed.
For all these reasons, I recommend to consider the publication of the present manuscript submitted to PLOS One after questions listed below have been addressed.

• Minor comments:
Title and running title should be revised to precise that sediment transport concerns only wind erosion.

Response: Thanks for this comment.  We have added 'aeolian' to the title and removed the running title.

ABSTRACT

l. 42-44: Not sure to understand the sentence. The word "and" should be removed, shouldn't it? Please revise.

Response:Thanks for catching this.  We have removed 'and'

INTRODUCTION

l. 62-65: the authors are imprecise. Wind speed is not an aerodynamic force. Here, the authors must refer to drag or lift. In the same way, soil moisture, soil crusts and vegetation structures are not forces that oppose soil particle removal. Here, the authors must refer to cohesive forces such as capillary forces or chemical binding forces. This sentence must be revised.

Response: Thanks for this comment. To address this comment, we have changed this sentence to the following. 'Broadly, wind erosion can be described as a balance of forces that remove particles from the surface (i.e., drag or lift associated with wind), and forces that oppose particle removal (e.g., capillary or chemical binding forces associated with soil moisture and organic material, etc.) [13–15]. '

MATERIALS AND METHODS

2.2 Sediment Transport Monitoring

l. 144-145: the authors chose to use two levels, 50 cm and 100 cm. In Flagg et al. (2014), it is stated that "We report sediment flux additively as the sum of sediment flux at 50 and 100 cm." Is it the same here? Please specify.

Response: Yes, we use the sum of 50 and 100 cm measurements, as in Flagg et al. (2014).

l. 149-151: in Flagg et al. (2014), it is stated that "Sediment samples were sometimes collected with de-ionized water if the traps were filled with water, and later freeze dried to remove the water. Samples were weighed to within 0.001 g (Denver Instruments Microscale, Denver, CO)". Here, another protocol was used. Please explain why or correct.

Response: Thanks, to clarify, we have changed this to the following: ' Consistent with Flagg et al. (2014), here we analyze the sum of sediment transport from 50 and 100 cm heights. In additionHere we analyze 11 years of continuous data, adding four years of data (2015-2018) to the Flagg et al. (2014) dataset and adding the 50 cm height flux to the data from Nauman et al. (2018). '

2.5 Soil Moisture Modeling

l. 191-192: why did the authors assign a value of 50 cm for plant height and a value of 1 for leaf area index. Please explain.

Response: We have added a comment and references to support these choices, as follow: 'which are consistent with personal observations and similar to previous observation for nearby systems [38,39].'

2.5 Data Analyses

Section number is incorrect and must be changed to 2.6

l. 212: the authors should refer to Table 1. Please verify.

Response: Thanks. We have removed section numbers.

l. 224: please define VIMP.

Response: We have changed 'variable importance (i.e., VIMP)' to 'variable importance (VIMP)'. Hopefully, this helps clarify.

RESULTS

3.1 Sediment Transport

l. 241: why did the authors average data at 50 cm and 100 cm whereas Flagg et al. (2014) summed the values? Please explain.

Response: Thanks for catching this. This was a type-o. We report the sum. We have changed this in the results.

l. 242: what is the interest to present the averaged value of sediment transport on 11 years and 52 sites considering the large scales of variations both in time (meteorological conditions) and space (different landscapes)? Please explain.

Response: This is a brief section where we just wanted to provide some simple measures to provide some context for the extent of sediment transport in the system. We feel these values may be helpful for researchers in other systems as a simple comparison or for modelers to constrain their values.

3.3 Random forest and regression tree models

Section number is incorrect and must be changed to 3.2

Response: Thanks. We have removed section numbers.

l. 273-274: the authors state from Figure 6 that “sediment flux increased as soil moisture at 30 cm increased.” Please, elaborate: give numbers, is it significant?
Response: Unfortunately, random forest model analyses don’t really allow this type of analysis. The model can explain a lot of variation in the data and can be expected to make good predictions, but the model is a bit of a ‘black box’ because it relies on averaging results from many regression trees. These partial dependence plots are a post-hoc approach to understanding what the model is doing. All we can say is that the model tends to predict more sediment flux as moisture at 30 cm increases and that this effect is much weaker than the relationship between wind speed and sediment transport.

DISCUSSION

l. 300-305: the authors should temper their words as the model they had designed is specific to the region where it was developed. Please, rephrase.
Response: Thanks, this is a good point. To clarify, we have added the following: ‘Whether or not the model can explain sediment flux in new landscapes remains to be determined and would require testing with new sediment flux data in new areas.’

REFERENCES

Response: We have overhauled to references, fixing several errors and matching journal format.

l. 408-411: this reference is misplaced and must be moved after Ballatyne et al. (2011).
Response: We have reordered references per Plos One style.

l. 419-421: Belnap et al. (2009) is not cited in the text. To be removed. On the other hand, Belnap et al. (1997) cited l. 365 is missing and must be added in this section.
Response: Belnap (2009) has been removed and Belnap (1997) has been added.

l. 446-447: Crawley and Nickling (2003) is not cited in the text. To be removed. On the other hand, Crawley et al. (2003) cited l. 342 is missing and must be added in this section.
Response: Crawley and Nickling (2003) is now referenced and included.

Flagg et al. (2023) cited l. 84 is missing and must be added in this section.
Response: This was a type-o and has been removed.

l. 461-462: Floyd and Gill (2011) is not cited in the text. To be removed.
Response: Floyd and Gill has been removed.

Fryrear (1986) cited l. 132-133 is missing and must be added in this section.
Response: Fryrear has been added.

l. 468-469: Gillette (1979) is not cited in the text. To be removed. On the other hand, Gillette et al. (1997) cited l. 75 is missing and must be added in this section.
Response:Gillette has been removed from the paper.

l. 474-476: Goldstein et al. (2010) is not cited in the text. To be removed.
Response:Goldstein has been removed.

l. 488-491: this reference is misplaced and must be moved after Hoover et al. (2021).
Response: the order has changed per Plos One style.

l. 492-494: please check the spelling of Hoffmann, written Hoffman when cited l. 102.
Response: This has been fixed.

l. 510-517: Lemos and Lutz (2010), Li et al. (2007), and Li et al. (2013) are not cited in the text. To be removed.
Response:These have been removed.

Miller et al. (2006) cited l. 160 is missing and must be added in this section.
Response: Miller has been removed from the paper.

l. 546-548: Saha (2003) is not cited in the text. To be removed.
Response: Removed.

Simunek et al. (2008) cited l. 181 is missing and must be added in this section.
Response: Simunek (2012 and 2016) are now included.

l. 558-559: Tchakerian (2014) is not cited in the text. To be removed.
Response: Removed.

l. 572-573: Webb & Strong (2011) and Webb et al. (2019) are not cited in the text. To be removed. On the other hand, Webb et al. (2011) cited l. 60 is missing and must be added in this section.
Response: Web (2011) and Webb (2018) are now included.

FIGURES
Figures 2 to 4: please add ticks on the y-axis for clarity.
Response: Thanks. We have added tick marks.

- Some typos:

l. 160: replace “eta al.” by “et al.”.
Response: removed.

l. 299: replace “Bruiec” by “Brieuc”.

Response: replaced.

**Reviewer #2:** The authors used 52 horizontal sediment flux and tested several approaches to compute sediment transport induced by wind and correlated with other variables such as soil moisture and land cover. They used random forest statistical analysis to improve results and made comparisons against a previous study using a regression tree.
The authors presented a good introduction, establishing what limitations they wanted to overcome and what goals they wanted to achieve. As a continuation of previous works, the authors have given the correct credits, but sometimes they forgot to provide more details of the methods. The results are consistent with the methods, but the presentation can be improved. Also, in the discussion part, more detais are needed to clarify the results.

Comments:
• Line 109: Include the reference to Fig 1 after “study area”,

Response: Thanks, we have added this reference.

• Line 122: Remove the link

Response: This was a link to the dataset, not the paper, but we have removed the link as suggested.

• Lines 191-192: You need to explain why you made those assumptions.
Response: We have added a comment and references to support these choices, as follow: ‘which are consistent with personal observations and similar to previous observation for nearby systems [38,39].’

• Line 198: “($F_{1,3813} = 3382$, $p < 0.05$, $R^2 = 0.47$).” what are these variables and what do they mean?
Response: These were the test statistics for a correlation between observed and predicted soil moisture values for the five sites for which we had observed soil moisture values. This was an ‘internal’ validation of our model. We have removed this sentence for clarity.

• Line 202: “While we primarily rely on random forest analyses”… It is important to give some brief explanation about these methods cited in this paragraph.

Response: Thanks, we have tried to clarify our statistical approaches as follows: ‘We primarily rely on random forest analyses (described below) because random forest has been found to be powerful approach for describing variation in a dataset when multiple covarying explanatory variables are available [26,27]. However, because random forest analyses complicated and difficult-to-interpret models, we also include univariate tests for differences in sediment transport associated with surface cover (Blackbrush, Grassland, Mancos, Pinyon/Juniper, Sagebrush, Saltbrush), grazing condition (No graze, Some graze, Grazed), and season. Univariate tests were used because they describe easy-to-interpret relationships between two variable. The effects of these conditions were testedbecause they were expected to be important and/or manageable. For these tests, we used completely randomized linear mixed models with surface cover, grazing condition, or season as the fixed effects.’

• Line 242: “of 6.96 ± 26.09”… What happens when we have 6.96 – 26.09? is it possible? What means this large SD?

Response: Large variations are typical for sediment flux. On calm days there will be no sediment flux, on windy days the flux can be very large. We want to include simple statistics about the dataset, so that other can place our values in context. For example, another system may have values of 2.0 +/- 5.0 g m-2 day-1 and another system may have values of 10.0 +/- 100 g m-2 day-1.

• Line 247: 3.1 and 3.3, what about 3.2?

Response: thanks for catching this.  We have removed section numbers to conform to Plos One style.

• Lines 262-265: what is the log sum? what is the partial dependence plots?

Response: Thanks for catching this. We neglected to describe the partial dependence statistic in the methods. This is a standard method in random forest analyses. To clarify, we have added the following to the Results: ‘Partial dependence plots show the relationship between a variable of interest and the random forest model output and can show linear or non-linear relationships [26]. Partial dependence plots show the log sum values which are the relative logit contribution of the variable on the class probability from the perspective of the model. Larger log sum values mean that the larger values of the predicted variable (i.e., sediment flux) are more likely in the model [27].’

• Lines 327-332: References! Is it possible that when soil moisture at 30cm is increasing the soil moisture at 5cm is decreasing? Do you also reflect that simulated soil moisture presented a low R2 and the uncertainty is higher than the correlation between sediment

transport and soil moisture?

Response: The low R2 between observed and predicted values largely reflects a timing difference. For example, if the model wetting front is a few days later than the observed wetting front, then it is easy to have a low correlation even though the general pattern of the wetting front is the same in both the modeled and observed data.

We think that our last explanation is the most likely – that soil moisture at 30 cm is associated with wet spring conditions when winds are highest. We have added this emphasis to the paper as follows: 'Perhaps most likely, it is possible that fast spring winds are associated with deeper soil percolation [52].'

• Figures 2-4: What is A, B, C D, etc.?

Response: In the figure legend, we note that '*Columns with a different letter are significantly different at the alpha = 0.05 level in a Tukey's HSD post-hoc test. PJ = piñon / juniper.*'

• Figure 6: I did not understand it. Figure 6 - wind (could be 'a') peak occurs for 2.5. You need to explain what are the axis putting units. Overall, the figures and their captions are not self-explanatory.

Response: We have elaborated on the Partial dependence plots in the Results. We have elaborated on each of the figure legends to make interpretation easier. For example, the Fig 6 legend is now: '**Fig 6. Partial dependence plots**. Plots show the relationship between variation in a single variable (x-axis) and the relative logit contribution of the variable on the class probability from the perspective of the model. In other words, large values (in the y-axis) mean that large values of sediment transport are more likely in the model.Values for peak wind, for example, show that sediment transport increases with wind speed, particularly when peak wind speeds exceed roughly 4 m $s^{-1}$. Note that axis values are not the same among variables.'

---

While revising your submission, please upload your figure files to the Preflight Analysis and Conversion Engine (PACE) digital diagnostic tool, https://pacev2.apexcovantage.com/. PACE helps ensure that figures meet PLOS requirements. To use PACE, you must first register as a user. Registration is free. Then, login and navigate to the UPLOAD tab, where you will find detailed instructions on how to use the tool. If you encounter any issues or have any questions when using PACE, please email PLOS at figures@plos.org. Please note that Supporting Information files do not need this step.

---

*In compliance with data protection regulations, you may request that we remove your personal registration details at any time. (Remove my information/details). Please contact the publication office if you have any questions.*
