# Supplementary material for: Season, wind speed, and seasonal rain are major drivers of a regional aeolian sediment transport model
Source: PLoS One. 2025 Sep 26;20(9):e0333166. doi: 10.1371/journal.pone.0333166 (PMC12468981; doi:10.1371/journal.pone.0333166)
Supplement: Attachment — Submitted filename: response to reviewers.docx [file pone.0333166.s004.docx]

PONE-D-25-20429R1

Season, wind speed, and seasonal rain are major drivers of a regional aeolian sediment transport model

PLOS ONE

Dear Dr. Kulmatiski,

Thank you for submitting your manuscript to PLOS ONE. After careful consideration, we feel that it has merit but does not fully meet PLOS ONE’s publication criteria as it currently stands. Therefore, we invite you to submit a revised version of the manuscript that addresses the points raised during the review process.

Please submit your revised manuscript by Oct 16 2025 11:59PM. If you will need more time than this to complete your revisions, please reply to this message or contact the journal office at [plosone@plos.org](mailto:plosone@plos.org). When you're ready to submit your revision, log on to https://www.editorialmanager.com/pone/ and select the 'Submissions Needing Revision' folder to locate your manuscript file.

Please include the following items when submitting your revised manuscript:

- A rebuttal letter that responds to each point raised by the academic editor and reviewer(s). You should upload this letter as a separate file labeled 'Response to Reviewers'.
- A marked-up copy of your manuscript that highlights changes made to the original version. You should upload this as a separate file labeled 'Revised Manuscript with Track Changes'.
- An unmarked version of your revised paper without tracked changes. You should upload this as a separate file labeled 'Manuscript'.

If you would like to make changes to your financial disclosure, please include your updated statement in your cover letter. Guidelines for resubmitting your figure files are available below the reviewer comments at the end of this letter.

If applicable, we recommend that you deposit your laboratory protocols in protocols.io to enhance the reproducibility of your results. Protocols.io assigns your protocol its own identifier (DOI) so that it can be cited independently in the future. For instructions see: [https://journals.plos.org/plosone/s/submission-guidelines#loc-laboratory-protocols](https://na01.safelinks.protection.outlook.com/?url=https%3A%2F%2Ftrack.editorialmanager.com%2FCL0%2Fhttps%3A%252F%252Fjournals.plos.org%252Fplosone%252Fs%252Fsubmission-guidelines%2523loc-laboratory-protocols%2F1%2F010f0199080fd338-c429da1e-d3e9-4e1c-86b6-c836fb8af71e-000000%2Fykhg6MomhohenSth-zbhSxm44aqlxSfjGogfUG00dfg%3D226&data=05%7C02%7C%7Cef653fb846e547da34f508dde9c0e2a7%7C84df9e7fe9f640afb435aaaaaaaaaaaa%7C1%7C0%7C638923736826674789%7CUnknown%7CTWFpbGZsb3d8eyJFbXB0eU1hcGkiOnRydWUsIlYiOiIwLjAuMDAwMCIsIlAiOiJXaW4zMiIsIkFOIjoiTWFpbCIsIldUIjoyfQ%3D%3D%7C0%7C%7C%7C&sdata=7nBWuzMCX9TMDXaa6KxCdRhzExbu%2Bg5Al%2FFF%2BqzBSWM%3D&reserved=0). Additionally, PLOS ONE offers an option for publishing peer-reviewed Lab Protocol articles, which describe protocols hosted on protocols.io. Read more information on sharing protocols at [https://plos.org/protocols?utm_medium=editorial-email&utm_source=authorletters&utm_campaign=protocols](https://na01.safelinks.protection.outlook.com/?url=https%3A%2F%2Ftrack.editorialmanager.com%2FCL0%2Fhttps%3A%252F%252Fplos.org%252Fprotocols%253Futm_medium%3Deditorial-email%2526utm_source%3Dauthorletters%2526utm_campaign%3Dprotocols%2F1%2F010f0199080fd338-c429da1e-d3e9-4e1c-86b6-c836fb8af71e-000000%2FSRM-ivFfTWAAaKOl8vPUVKmRu9EO-5NF2sqIn_q52kM%3D226&data=05%7C02%7C%7Cef653fb846e547da34f508dde9c0e2a7%7C84df9e7fe9f640afb435aaaaaaaaaaaa%7C1%7C0%7C638923736826697132%7CUnknown%7CTWFpbGZsb3d8eyJFbXB0eU1hcGkiOnRydWUsIlYiOiIwLjAuMDAwMCIsIlAiOiJXaW4zMiIsIkFOIjoiTWFpbCIsIldUIjoyfQ%3D%3D%7C0%7C%7C%7C&sdata=4uoSRWpC1sUncg1K%2BBkFAqq3bVC9utDcijLHj5jZR%2B0%3D&reserved=0).

We look forward to receiving your revised manuscript.

Kind regards,

Jamil Alexandre Ayach Anache
Academic Editor
PLOS ONE

Journal Requirements:

1. If the reviewer comments include a recommendation to cite specific previously published works, please review and evaluate these publications to determine whether they are relevant and should be cited. There is no requirement to cite these works unless the editor has indicated otherwise. 

Please review your reference list to ensure that it is complete and correct. If you have cited papers that have been retracted, please include the rationale for doing so in the manuscript text, or remove these references and replace them with relevant current references. Any changes to the reference list should be mentioned in the rebuttal letter that accompanies your revised manuscript. If you need to cite a retracted article, indicate the article’s retracted status in the References list and also include a citation and full reference for the retraction notice.

Additional Editor Comments:

Please, check Reviewer #1 final comments.


[Note: HTML markup is below. Please do not edit.]

Reviewers' comments:

Reviewer's Responses to Questions

**Comments to the Author**

1. If the authors have adequately addressed your comments raised in a previous round of review and you feel that this manuscript is now acceptable for publication, you may indicate that here to bypass the “Comments to the Author” section, enter your conflict of interest statement in the “Confidential to Editor” section, and submit your "Accept" recommendation.

Reviewer #1: (No Response)

Reviewer #2: All comments have been addressed

2. Is the manuscript technically sound, and do the data support the conclusions?

The manuscript must describe a technically sound piece of scientific research with data that supports the conclusions. Experiments must have been conducted rigorously, with appropriate controls, replication, and sample sizes. The conclusions must be drawn appropriately based on the data presented.

Reviewer #1: Yes

Reviewer #2: Yes

3. Has the statistical analysis been performed appropriately and rigorously?

Reviewer #1: Yes

Reviewer #2: Yes

4. Have the authors made all data underlying the findings in their manuscript fully available?

The [PLOS Data policy](https://na01.safelinks.protection.outlook.com/?url=http%3A%2F%2Ftrack.editorialmanager.com%2FCL0%2Fhttp%3A%252F%252Fwww.plosone.org%252Fstatic%252Fpolicies.action%2523sharing%2F1%2F010f0199080fd338-c429da1e-d3e9-4e1c-86b6-c836fb8af71e-000000%2FUQLmlin8jbmHz61zGE1lLLZSM3OTWFz6z5bmKEJJlGA%3D226&data=05%7C02%7C%7Cef653fb846e547da34f508dde9c0e2a7%7C84df9e7fe9f640afb435aaaaaaaaaaaa%7C1%7C0%7C638923736826709775%7CUnknown%7CTWFpbGZsb3d8eyJFbXB0eU1hcGkiOnRydWUsIlYiOiIwLjAuMDAwMCIsIlAiOiJXaW4zMiIsIkFOIjoiTWFpbCIsIldUIjoyfQ%3D%3D%7C0%7C%7C%7C&sdata=zc4bK%2BDWR20q4T5DKDH0XwQ%2FgmgeUAWrlcWy8TRbPr4%3D&reserved=0) requires authors to make all data underlying the findings described in their manuscript fully available without restriction, with rare exception (please refer to the Data Availability Statement in the manuscript PDF file). The data should be provided as part of the manuscript or its supporting information, or deposited to a public repository. For example, in addition to summary statistics, the data points behind means, medians and variance measures should be available. If there are restrictions on publicly sharing data—e.g. participant privacy or use of data from a third party—those must be specified.

Reviewer #1: Yes

Reviewer #2: Yes

5. Is the manuscript presented in an intelligible fashion and written in standard English?

PLOS ONE does not copyedit accepted manuscripts, so the language in submitted articles must be clear, correct, and unambiguous. Any typographical or grammatical errors should be corrected at revision, so please note any specific errors here.

Reviewer #1: Yes

Reviewer #2: Yes

6. Review Comments to the Author

Please use the space provided to explain your answers to the questions above. You may also include additional comments for the author, including concerns about dual publication, research ethics, or publication ethics. (Please upload your review as an attachment if it exceeds 20,000 characters)

Reviewer #1: I thank the authors who answered all my comments convincingly.
However, there is still a mistake in the manuscript in l. 146 and in l. 248. Indeed, in Flagg et al. (2014), it is stated that “We report sediment flux additively as the sum of sediment flux at 50 and 100 cm.” In the answer the authors made to one of my question in the first round of review, they confim that they used the sum of 50 and 100 cm measurements, as in Flagg et al. (2014). Unfortunately, in l. 146 and l. 248, it is still written that the authors used the average of sediment transport from 50 and 100 cm heights. This must be corrected before the manuscript is suitable for publication in PLOS One.

Response: Thanks for catching this oversight. We have clarified that the sum was calculated and reported at lines 146 (155) and 248 (246)

Reviewer #2: (No Response)

7. PLOS authors have the option to publish the peer review history of their article ([what does this mean?](https://na01.safelinks.protection.outlook.com/?url=https%3A%2F%2Ftrack.editorialmanager.com%2FCL0%2Fhttps%3A%252F%252Fjournals.plos.org%252Fplosone%252Fs%252Feditorial-and-peer-review-process%2523loc-peer-review-history%2F1%2F010f0199080fd338-c429da1e-d3e9-4e1c-86b6-c836fb8af71e-000000%2FOCWy2FqxfuaqrDcqWXVk5x2Yhi0SMlG4wnvoNid6ioM%3D226&data=05%7C02%7C%7Cef653fb846e547da34f508dde9c0e2a7%7C84df9e7fe9f640afb435aaaaaaaaaaaa%7C1%7C0%7C638923736826720484%7CUnknown%7CTWFpbGZsb3d8eyJFbXB0eU1hcGkiOnRydWUsIlYiOiIwLjAuMDAwMCIsIlAiOiJXaW4zMiIsIkFOIjoiTWFpbCIsIldUIjoyfQ%3D%3D%7C0%7C%7C%7C&sdata=atGisGUfIO76QAvvRUzTon3HpXRb3TVlcc2paQc3w3I%3D&reserved=0)). If published, this will include your full peer review and any attached files.


If you choose “no”, your identity will remain anonymous but your review may still be made public.


**Do you want your identity to be public for this peer review?** For information about this choice, including consent withdrawal, please see our [Privacy Policy](https://na01.safelinks.protection.outlook.com/?url=https%3A%2F%2Ftrack.editorialmanager.com%2FCL0%2Fhttps%3A%252F%252Fwww.plos.org%252Fprivacy-policy%2F1%2F010f0199080fd338-c429da1e-d3e9-4e1c-86b6-c836fb8af71e-000000%2F2XpXrkngNRJWokRJzEqptIQPIRGBcqQ0THg2Li1utSM%3D226&data=05%7C02%7C%7Cef653fb846e547da34f508dde9c0e2a7%7C84df9e7fe9f640afb435aaaaaaaaaaaa%7C1%7C0%7C638923736826729615%7CUnknown%7CTWFpbGZsb3d8eyJFbXB0eU1hcGkiOnRydWUsIlYiOiIwLjAuMDAwMCIsIlAiOiJXaW4zMiIsIkFOIjoiTWFpbCIsIldUIjoyfQ%3D%3D%7C0%7C%7C%7C&sdata=ta9EmMqhaa9xQ93OvkFHc8Sc04nZQwSnf1Plzb%2BhQss%3D&reserved=0).

Reviewer #1: No

Reviewer #2: No

[NOTE: If reviewer comments were submitted as an attachment file, they will be attached to this email and accessible via the submission site. Please log into your account, locate the manuscript record, and check for the action link "View Attachments". If this link does not appear, there are no attachment files.]

While revising your submission, please upload your figure files to the Preflight Analysis and Conversion Engine (PACE) digital diagnostic tool, [https://pacev2.apexcovantage.com/](https://na01.safelinks.protection.outlook.com/?url=https%3A%2F%2Ftrack.editorialmanager.com%2FCL0%2Fhttps%3A%252F%252Fpacev2.apexcovantage.com%252F%2F1%2F010f0199080fd338-c429da1e-d3e9-4e1c-86b6-c836fb8af71e-000000%2FxRJqfnGLtBCiMFVTFJMxi5tXp8wjrV7imv8VgMUmZaY%3D226&data=05%7C02%7C%7Cef653fb846e547da34f508dde9c0e2a7%7C84df9e7fe9f640afb435aaaaaaaaaaaa%7C1%7C0%7C638923736826738273%7CUnknown%7CTWFpbGZsb3d8eyJFbXB0eU1hcGkiOnRydWUsIlYiOiIwLjAuMDAwMCIsIlAiOiJXaW4zMiIsIkFOIjoiTWFpbCIsIldUIjoyfQ%3D%3D%7C0%7C%7C%7C&sdata=FeP0DTlbgdStHgJIicLEt3DyLjYAMVCWbfvozOdeXXc%3D&reserved=0). PACE helps ensure that figures meet PLOS requirements. To use PACE, you must first register as a user. Registration is free. Then, login and navigate to the UPLOAD tab, where you will find detailed instructions on how to use the tool. If you encounter any issues or have any questions when using PACE, please email PLOS at [figures@plos.org](mailto:figures@plos.org). Please note that Supporting Information files do not need this step.
